# VapC21 Toxin Contributes to Drug-Tolerance and Interacts With Non-cognate VapB32 Antitoxin in *Mycobacterium tuberculosis*

**DOI:** 10.3389/fmicb.2020.02037

**Published:** 2020-09-11

**Authors:** Arun Sharma, Gopinath Chattopadhyay, Pankaj Chopra, Munmun Bhasin, Chandrani Thakur, Sakshi Agarwal, Shahbaz Ahmed, Nagasuma Chandra, Raghavan Varadarajan, Ramandeep Singh

**Affiliations:** ^1^Tuberculosis Research Laboratory, Translational Health Science and Technology Institute, Faridabad, India; ^2^Molecular Biophysics Unit, Indian Institute of Science, Bengaluru, India; ^3^Department of Biochemistry, Indian Institute of Science, Bengaluru, India; ^4^Jawaharlal Nehru Centre for Advanced Scientific Research, Bengaluru, India

**Keywords:** *Mycobacterium tuberculosis*, toxin-antitoxin system, ribonuclease, drug tolerance, cross talk

## Abstract

The prokaryotic ubiquitous Toxin-antitoxin (TA) modules encodes for a stable toxin and an unstable antitoxin. VapBC subfamily is the most abundant Type II TA system in *M. tuberculosis* genome. However, the exact physiological role for most of these Type II TA systems are still unknown. Here, we have comprehensively characterized the VapBC21 TA locus from *M. tuberculosis*. The overexpression of VapC21 inhibited mycobacterial growth in a bacteriostatic manner and as expected, growth inhibition was abrogated upon co-expression of the cognate antitoxin, VapB21. We observed that the deletion of *vapC21* had no noticeable influence on the *in vitro* and *in vivo* growth of *M. tuberculosis*. Using co-expression and biophysical studies, we observed that in addition to VapB21, VapC21 is also able to interact with non-cognate antitoxin, VapB32. The strength of interaction varied between the cognate and non-cognate TA pairs. The overexpression of VapC21 resulted in differential expression of approximately 435 transcripts in *M. tuberculosis.* The transcriptional profiles obtained upon ectopic expression of VapC21 was similar to those reported in *M. tuberculosis* upon exposure to stress conditions such as nutrient starvation and enduring hypoxic response. Further, VapC21 overexpression also led to increased expression of WhiB7 regulon and bacterial tolerance to aminoglycosides and ethambutol. Taken together, these results indicate that a complex network of interactions exists between non-cognate TA pairs and VapC21 contributes to drug tolerance *in vitro*.

## Introduction

Tuberculosis (TB) with an incident rate and deaths of 10 million and 1.4 million individuals, respectively, was the leading cause of death worldwide among infectious diseases in 2018 (Glaziou et al., 2018). *M. tuberculosis* is a highly successful pathogen because of its ability to subvert host antimicrobial pathways and persist in host tissues (Ehrt and Schnappinger, 2009). However, the exact molecular switches that enable *M. tuberculosis* to slow down metabolism and enter into dormant or latent state still remains unknown. Toxin-antitoxin (TA) systems, initially referred as addiction systems, are auto-regulatory operon encoding for a labile antitoxin and stable toxin (Lobato-Marquez et al., 2016; Page and Peti, 2016; Harms et al., 2018; Holden and Errington, 2018; Kang et al., 2018). The toxin-mediated growth arrest is mostly bacteriostatic, reversible and regulated by the expression levels of cognate antitoxins (Muthuramalingam et al., 2016). The characterized toxins most likely inhibit bacterial growth by targeting an essential cellular processes such as protein synthesis or cell wall synthesis or cell division or DNA replication (Lobato-Marquez et al., 2016; Harms et al., 2018). TA systems have been broadly characterized into 6 different groups based on the nature and neutralization mechanisms of antitoxins (Schuster and Bertram, 2013; Page and Peti, 2016). Among these, Type II is the most well characterized TA family where the antitoxin negates the activity of cognate toxins by forming protein-protein complexes. The genome of *M. tuberculosis* encodes for a large repertoire of Type II TA systems such as *mazEF*, *relBE*, *higBA*, *parDE*, and *vapBC* (Pandey and Gerdes, 2005; Ramage et al., 2009; Akarsu et al., 2019; Tandon et al., 2019b). Using inducible expression systems, it has been reported that ectopic expression of the majority of these toxins inhibits bacterial growth in a bacteriostatic manner (Gupta, 2009; Ramage et al., 2009; Winther et al., 2016; Agarwal et al., 2018). Among these, MazF toxins cleaves mRNA in a sequence specific but ribosome independent manner and contribute cumulatively to the ability of *M. tuberculosis* to establish infection in host tissues (Zhang et al., 2003; Cook et al., 2013; Tiwari et al., 2015). In another study, it has been reported that deletion and overexpression of *M. tuberculosis* RelE toxins resulted in decreased and increased number of drug-tolerant populations, respectively, *in vitro* (Singh et al., 2010).

The virulence associated protein B and C, VapBC TA system comprises of VapB antitoxin and VapC toxin (Pandey and Gerdes, 2005; Ramage et al., 2009; Tandon et al., 2019b). The VapB antitoxin possesses DNA binding and toxin binding domains at its amino- and carboxy terminus, respectively (Bendtsen and Brodersen, 2017). VapC toxins are characterized by the presence of an amino-terminal PilT-domain. This domain consists of a conserved quartet of acidic and invariant Ser/Thr amino acid residues responsible for coordinating divalent ions (Arcus et al., 2011). Several studies have shown that both *vapB* antitoxins and *vapC* toxins are differentially expressed in stress conditions that *M. tuberculosis* might encounter during infection (Ramage et al., 2009; Keren et al., 2011; Agarwal et al., 2018). The cellular targets for these ribonucleases have also been extensively characterized and include tRNA32^Gln–CTG^, tRNA3^Leu–CAG^, tRNA21^Cys–GCA^, tRNA^fmet^, tRNA25^Ser–TGA^, tRNA28^Ser–CGA^, and tRNA7^Trp–CCA^ (Winther and Gerdes, 2011; Sharp et al., 2012; Cruz et al., 2015; Winther et al., 2016; Cintron et al., 2019). The crystal structures for some of these VapBC TA systems have been solved with the following PDB codes; 3H87 (Rv0300-Rv0301), 3DB0 (Rv0626-Rv0627), 4CHG (Rv2009-Rv2010), 4XGQ (Rv0623-Rv0624), 5X3T (Rv0581-Rv0582), and 6A7V (Rv1560-Rv1561) (Miallau et al., 2009; Min et al., 2012; Das et al., 2014; Lee et al., 2015; Kang et al., 2017; Deep et al., 2018). Additionally, the structure of VapC toxin, Rv2549c that cleave 23S rRNA at the sarcin-ricin loop have also been solved (Winther et al., 2013; Deep et al., 2017). In our earlier reports, we have shown VapBC3, VapBC4, and VapBC11 are essential for *M. tuberculosis* to establish infection in guinea pigs (Agarwal et al., 2018; Deep et al., 2018). However, *M. tuberculosis* strain deficient in *vapC28* did not exhibit a growth defect in the guinea pig model of infection (Agarwal et al., 2018).

Here, we have performed a detailed functional and biochemical characterization of VapBC21 (Rv2757c-Rv2758c) TA system from *M. tuberculosis*. VapBC21 TA complex is absent in *M. smegmatis* but present in the genome sequences of members belonging to *M. tuberculosis* complex (Ramage et al., 2009; Tandon et al., 2019b). Although, the crystal structure of VapC21 is available, the cellular target for VapC21 is still unknown (Jardim et al., 2016). In the present study, we demonstrate that inducible expression of VapC21 inhibited *M. smegmatis* growth in a bacteriostatic manner. We show that VapC21 is dispensable for *M. tuberculosis* survival in different stress conditions and in mice model of infection. Using growth assays, we demonstrate that in addition to VapB21, VapB32 coexpression was also able to abrogate the growth inhibition associated with VapC21 expression in *M. smegmatis*. We also performed surface plasmon resonance (SPR) experiments and size exclusion chromatography-multi angle light scattering (SEC-MALS) to determine the relative stabilities and binding affinities of cognate and non-cognate TA pairs. The transcription profiles obtained upon VapC21 overexpression overlapped with the profiles obtained in *M. tuberculosis* upon exposure to different stress conditions. VapC21 overexpression also increased the survival of *M. smegmatis* upon exposure to aminoglycoside and ethambutol. Taken together, we have performed a detailed biochemical and functional characterization of the VapBC21 TA system from *M. tuberculosis*.

## Materials and Methods

### Culture Condition and Generation of Mutant and Complemented Strains

The list of strains and plasmids used in the study are shown in Supplementary Table S1. *E. coli* strains were cultured in either Luria Bertani Broth (LB) or Terrific Broth (TB) with shaking at 200 rpm at 37°C. The mycobacterial strains were cultured in Middlebrook 7H9 medium containing 0.2% glycerol, 0.05% Tween-80 and supplemented with 1× ADS at 200 rpm at 37°C as previously described (Singh et al., 2013). The *E. coli* and mycobacterial cultures were plated on LB agar and Middlebrook 7H11 Agar supplemented with 1× OADS at 37°C, respectively. The following antibiotics were added to the medium as and when required; 25 μg/ml kanamycin for both *E. coli* and mycobacteria, 50 μg/ml ampicillin and 10 μg/ml tetracycline for *E. coli*, and 150 μg/ml and 50 μg/ml hygromycin for *E. coli* and mycobacteria, respectively. Unless mentioned otherwise, all chemicals used in the study were procured from Sigma Aldrich, Merck. MIC_99_ determination assays of *M. tuberculosis* strains against various drugs were performed as previously described (Kidwai et al., 2017).

The *M. tuberculosis* Erdman strain lacking the ribonuclease activity associated with VapC21 was constructed using temperature sensitive mycobacteriophages as previously described (Bardarov et al., 2002). Briefly, approximately 800 bp upstream and downstream regions flanking the *vapC21* gene were cloned into cosmid, pYUB854. The recombinant cosmid, pYUB854-Δ*vapC21* was *Pac*I digested and packaged into phagemid, phAE159 using MaxPlax^TM^ Lambda Packaging Extract. The recombinant cosmid, phAE159-Δ*vapC21* was introduced into electrocompetent cells of *M. smegmatis* to generate temperature sensitive mycobacteriophages. The mutant strain of *M. tuberculosis* was constructed using these high-titre mycobacteriophages. The replacement of the VapC21 open reading frame with the hygromycin resistance gene in *M. tuberculosis* genome was confirmed by Southern blot. For construction of the complemented strain, *vapC21* was PCR amplified, cloned into pVV16 under the transcriptional control of the *hsp65* promoter. The resulting plasmid, pVV16-*vapC21* was introduced into the electrocompetent cells of Δ*vapC21* strain.

### *In vitro* Stress and *in vivo* Experiments

The growth patterns of both wild type and mutant strain were compared by measuring OD_600nm_ at regular intervals and CFU analysis. The colony morphology and ability of these strains to form biofilms was determined as previously described (Arora et al., 2018; Tiwari et al., 2019). The effect of deletion of *vapC21* on the survival of *M. tuberculosis* was evaluated in the following conditions: oxidative stress (5 mM H_2_O_2_ for 3 days), nitrosative stress (5 mM NaNO_2_, pH-5.2 for 3 days), 0.25% SDS for 3 days, 2.5 mg/ml lysozyme for 3 days, nutrient starvation (1× Tris buffered saline, 1× TBS with 0.05% Tween-80 for 7 and 14 days), drugs (isoniazid, rifampicin, and levofloxacin for 14 days) as previously described (Arora et al., 2018; Tiwari et al., 2019). For bacterial enumeration, at designated time points, 10-fold serial dilutions were prepared and plated on Middlebrook 7H11 medium 37°C for 3–4 weeks.

The animal experiments were performed in accordance with guidelines recommended by the CPCSEA, Govt. of India and appropriate approvals were obtained from the animal ethics committee of Translational Health Science and Technology Institute. Prior to infection, mid-log phase cultures of *M. tuberculosis* were harvested, washed and single-cell suspensions were prepared. Six to eight week old mice were infected with either wild type or Δ*vapC21* or Δ*vapC21-CT* strains via aerosol route, resulting into implantation of 50-100 bacilli in lung tissues. The lung bacillary loads of infected animals at 4- and 8-weeks post-infection was determined by homogenizing tissues in 2.0 ml of normal saline and 10-fold serial dilutions were plated on Middlebrook 7H11 plates at 37°C for 3–4 weeks.

### Coexpression and Growth Inhibition Studies

For growth inhibition studies in *M. smegmatis, vapC21* was PCR amplified and cloned into the anyhydrotetracycline (Atc) inducible integrative vector, pTetR-Int (Ehrt et al., 2005; Agarwal et al., 2018). For coexpression studies, various antitoxins were PCR amplified and cloned into acetamide inducible vector, pLAM12 (van Kessel et al., 2008). The final recombinant constructs were confirmed by DNA sequencing. For growth assays, recombinant *M. smegmatis* strains were grown until early-log phase (OD_600nm_ ∼ 0.2) and expression of toxin and antitoxin was induced by the addition of 50 ng/ml Atc and 0.2% acetamide, respectively. The growth of different strains was determined by either measuring absorbance (OD_600nm_) at regular intervals or by spotting assays. For spotting assays, at 12 h post-induction, an aliquot was removed and 10-fold serial dilutions were spotted on Middlebrook 7H11 plates at 37°C for 2–3 days.

### Cloning, Protein Expression, and Purification

For expression and purification of the VapBC21 complex, *vapB21* (Rv2758c) and *vapC21* (Rv2757c) were cloned individually into the pET-Duet vector. In order to purify toxin and antitoxins individually, the genes encoding either *vapC21* (Rv2757c) or *vapB21* (Rv2758c), *vapB3* (Rv0550c), *vapB4* (Rv0596c), *vapB26* (Rv0581), or *vapB32* (Rv1113) were PCR amplified and cloned into pET15b. The recombinant BL-21 (λDE3, plysE) strains were grown in TB medium until an OD_600nm_ ∼ 0.6 and protein expression was induced upon the addition of 1.0 mM isopropyl β-D-1-thiogalactopyranoside. The cultures were induced for 5 h at 37°C in the case of VapBC21 complex, 16 h at 20°C for various antitoxins and VapC21 protein. The induced cultures were harvested, resuspended in lysis buffer (10 mM HEPES, pH-8.0, 100 mM NaCl, 100 mM arginine, 10% glycerol and protease inhibitor) and lysed by sonication. The clarified lysates were incubated with 2 ml Ni-Sepharose resin with end-to-end mixing for 4 h and recombinant protein was purified as per manufacturer recommendations. The purified proteins were subjected to 15% Tricine SDS-PAGE, concentrated and stored in storage buffer (10 mM HEPES, 100 mM NaCl, 100 mM arginine, 10% glycerol, 500 mM imidazole) at −80°C until further use. Further, a C-terminal VapB21 peptide (residues 69–108), synthesized from GeneScript was also used in the study.

### NanoDSF, SEC-MALS, and SPR Studies

Thermal unfolding experiments were performed using nanoDSF of different proteins as previously described (Teale and Weber, 1957; Bruce et al., 2019; Chattopadhyay and Varadarajan, 2019; Magnusson et al., 2019). nanoDSF, measures the changes in the fluorescence of the intrinsic fluorophores tryptophan and tyrosine upon protein unfolding in a label free manner. The excitation was performed at 280 nm and emission at 330 and 350 nm and F350/330 was calculated for analysis. Briefly, 10 μM of each protein was filled in capillaries and subjected to heating from 20°C to 90°C, with a ramp rate of 1°C/min. The first derivative of F350/330 was plotted as a function of temperature, to determine the apparent T_m_ for different proteins. For SEC-MALS experiments, 100 μg of VapB antitoxins or VapC toxin or VapBC complexes were injected and separated using pre-equilibrated Superdex-200 analytical gel filtration column connected with in-line UV (SHIMADZU), MALS (mini DAWN TREOS, Wyatt Technology orp) and refractive index detectors (WATERS24614) to determine molecular weight, oligomeric state and aggregation. The equilibration of the instrument was performed in buffer containing 10 mM HEPES, pH-8.0, 100 mM NaCl, 100 mM arginine and 500 mM imidazole. The data was collected and analyzed using ASTRA^TM^ software as previously described (Kesavardhana et al., 2017). The SPR experiments were performed using a Biacore 3000 optical biosensor at 25°C. The dialyzed VapC21 protein was immobilized on the surface of a CM5 chip using a standard amine coupling kit as per the manufacturer’s recommendations. All experiments included an activated and deactivated sensor surface (without VapC21) as negative control. The dialyzed VapB proteins in the concentration range of 25 nM to 5 μM were run across each sensor surface at a flow rate of 30 μL/min in 1× PBS (pH-7.4) running buffer containing 0.005% Tween – 20. The regeneration of the sensor surface between runs was performed by washing the sensor chip twice with a solution of 4 M MgCl_2_ for 10–30 s at a flow rate 30 μL/min. The binding curve data for each interaction was normalized, fitted to a simple 1:1 Langmuir interaction model using BIA EVALUATION 3.1 software and various kinetic parameters were determined.

### RNA Sequencing Experiments

In the case of the overexpression strain, total RNA was isolated from *M. tuberculosis* harboring either pTetR-int or pTetR-Int-*vapC21* grown until an OD_600nm_ ∼ 0.2 and the expression was induced with 50 ng/ml anhydrotetracycline for 24 h. In order to determine the effect of deletion of *vapC21* on *M. tuberculosis* physiology, parental and mutant strains were grown until mid-log phase (OD_600nm_ ∼ 0.8). For RNA-seq experiments, the bacterial cultures were harvested, washed, and total RNA was isolated using the TRIzol method (Singh et al., 2013). The isolated RNA was subjected to DNase I treatment, cDNA libraries were prepared from rRNA depleted samples and sequenced using a Illumina HiSeq platform at Aggrigenome labs Pvt. Ltd. as previously described (Tiwari et al., 2019). The data obtained from the mutant strain was analyzed as previously mentioned (Tiwari et al., 2019). The quality of the raw data obtained from the overexpression strain was further assessed using FastQCv 0.11.8. Subsequently, the adaptor sequences were removed from QC-passed reads using Trim Galore v0.6.4 and the cleaned reads were subjected to further analysis. The indexing of the reference genomes was performed using Bowtie2 and the trimmed paired-end reads were aligned to either *M. tuberculosis* Erdman or H_37_Rv genome with TopHat v2.1.1 using default parameter settings for read obtained from mutant and overexpression strain, respectively (Langmead and Salzberg, 2012; Kim et al., 2013). The reference genome was downloaded from ENSEMBL Database using the FTP download. The resulting mapped files in SAM format were converted to BAM format using SAMtools v1.9 (Li et al., 2009). The assembly of transcripts was performed using Cufflinks v2.2.1, the separately assembled transcripts were merged into a cohesive set using Cuffmerge and the differentially expressed transcripts were identified using Cuffdiff (Trapnell et al., 2010). This provided an average expression value for an individual gene in the form of fragment per kilobase of transcript per million mapped reads (FPKM) as the output. The fold change for each transcript was calculated relative to the vector control by taking a ratio of their corresponding FKPM values. The Cuffdiff output of gene counts was subsequently analyzed for differential gene expression using CummeRbund (R-package) software. The volcano plots for the identified transcripts were plotted using the basic plot function in R software.

### qPCR Analysis

For qPCR analysis, 1 μg of DNase I treated mRNA was subjected to cDNA preparation using Superscript III reverse transcriptase. The synthesized cDNA was used as a template for qPCR using gene specific primers and SYBR Green mix. The data obtained was normalized to the transcript levels obtained for *sigA*, housekeeping gene as described previously (Singh et al., 2013).

### Network Analysis

The genome-wide knowledge-based protein-protein interaction (PPI) for *M. tuberculosis* was used as a base network to generate a condition specific network (Mishra et al., 2017). The network is directed in most part, where direction information was obtained from databases such as KEGG and STRING (Kanehisa and Goto, 2000; von Mering et al., 2003). In the network, proteins are represented as nodes while the interactions between proteins are represented as edges. The FKPM values for all the genes were used for assigning weights to corresponding nodes and edges in order to generate a condition-specific PPI network. The node weight (NW) values were calculated for each node in the network corresponding to the fold-change (FC) values using the following equation.

$$NW_{i}=\frac{\left[FPKM_{Test}\right]}{\left[FPKM_{Control}\right]}$$

where, *i* denotes the node in the network. The node weight values were used to calculate the edge weight (EW) values using the following equation.

$$EW_{i,j}=\frac{1}{\sqrt{NW_{i}*NW_{j}}}$$

where, *i* and *j* denotes nodes present in an edge.

The shortest paths between all the nodes in the weighted and directed network were computed using Dijkstra’s algorithm. The algorithm computes minimum weight shortest paths, in which each path begins from a source node and ends with a sink node, through interacting proteins, choosing the least-cost edge in every step. The edge cost values were used as an input for calculating all vs. all shortest paths in each condition using Zen^1^. This was followed by computing a top-response network using a previously described network analysis pipeline (Sambarey et al., 2013; Sambaturu et al., 2016). Instead of analyzing all the paths, a subnetwork comprising the top-ranking paths were considered to constitute the top-response network. The path score was computed as a summation of the edge weights constituting the path and normalized with the path length. The resultant shortest paths were ranked based on their normalized path score using a percentile approach, with paths having a lower path score given a higher rank. Thereafter, top 0.05 percentile of highest-ranked paths were pooled and viewed as a network. The generated response networks were visualized using Cytoscape 3.7.1 (Shannon et al., 2003).

### Drug Persistence Assays in VapC21 Overexpressing Strains of *M. smegmatis*

The concentration of drugs used for the drug tolerance experiments were as follows: 0.78 μM amikacin, 0.2 μM streptomycin and 1.56 μM ethambutol. For drug tolerance experiments, the expression of VapC21 in the overexpression strain was induced by the addition of 50 ng/ml Atc. The induced cultures were diluted to an OD_600nm_ of 0.2 and subsequently exposed to various drugs. After 12 h post-exposure, an aliquot was removed and CFU enumeration was performed. For bacterial enumeration, 10-fold serial dilutions were prepared and plated on Middlebrook 7H11 plates. Percent survival was calculated from the obtained CFU/ml after incubation with the drug divided by the CFU/ml obtained at time zero.

### Statistical Analysis

Differences between groups were determined by paired (two-tailed) *t* test and were considered significant at a *P* value of < 0.05. GraphPad Prism version 8 (GraphPad Software Inc., CA, United States) was used for statistical analysis and the generation of graphs.

## Results

### Overexpression of VapC21 Induces Bacteriostasis in *M. smegmatis*

**FIGURE 1:**
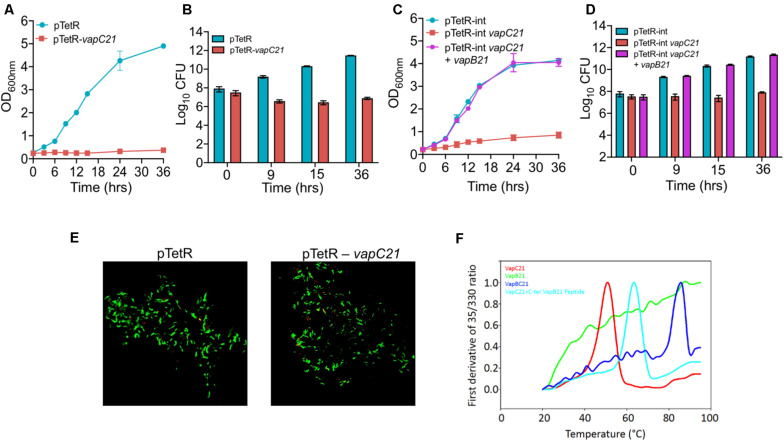
Functional characterization of VapBC21 TA system from *M. tuberculosis*. **(A,B)** The recombinant *M. smegmatis* strains were growth until OD_600nm_ ∼ 0.2 and the expression of toxin was induced by the addition of 50 ng/ml Atc. The effect of overexpression of VapC21 on the growth of *M. smegmatis* was determined either by measuring OD_600nm_ **(A)** or by CFU enumeration **(B)**. **(C,D)** In our co-expression experiment, the expression of toxin and antitoxin was induced by the addition of 50 ng/ml Atc and 0.2% acetamide, respectively. The growth of various recombinant *M. smegmatis* strains was monitored by measuring OD_600nm_ or by CFU enumeration. The data shown in panels **(A–D)** is obtained from three independent experiments. **(E)** For live-dead staining, single cell suspension was prepared and stained with a mixture of propidium iodide and SYTO-9. Images were viewed using FV3000 confocal microscope (Olympus, Japan) at 100× magnification. **(F)** Thermal stability of 10 μM of VapB21 (green), VapC21 (red), VapBC21 complex (blue), and 20 μM of VapB21 peptide incubated with 10 μM of VapC21 (cyan) was determined by nanoDSF.

VapBC family is the most abundant family of Type II TA systems in *M. tuberculosis* and its genome encodes for approximately 50 VapBC homologs (Ramage et al., 2009; Tandon et al., 2019b). VapC toxins inhibit mycobacterial growth by cleaving either mRNA, rRNA, or tRNA and their activity is neutralized by the levels of their cognate antitoxin (Winther et al., 2013; Cruz et al., 2015; Cintron et al., 2019). In our earlier studies, using the Atc based expression vector, pTetR, we have functionally characterized VapC toxins in *M. bovis* BCG as either inactive, moderately active or highly active proteins (Agarwal et al., 2018). The toxins were classified as highly active, where no significant increase in absorbance was observed upon induction in liquid cultures. This activity associated with the highly active toxins might be attributed to either increased expression levels or endoribonuclease activity or the essentiality of their cellular targets (Agarwal et al., 2018). In the present study, we have functionally and biochemically characterized VapC21 (Rv2757c) that belongs to the highly active VapC toxins group. Using episomal and integrative inducible vectors, we report that ectopic expression of VapC21 severely inhibited *M. smegmatis* growth in a bacteriostatic manner and this was abrogated in the presence of its cognate antitoxin, VapB21 (Figures 1A–D). The bacterial counts were reduced by approximately 45-, 615-, and 1500-fold in strains harboring an integrative copy of VapC21 at 9, 15, and 36 h, respectively post-Atc induction in comparison to parental strain (Figure 1D). In agreement with the absorbance based data, the reduction in viable bacterial counts was reversed by the co-expression of their cognate antitoxin, VapB21 (Figures 1C,D). As expected, LIVE-DEAD cell viability kit revealed that the bacilli overexpressing VapC21 were as viable as the parental strain (Figure 1E). Further, to determine the effect of VapC21 overexpression on cellular morphology of *M. smegmatis*, DAPI-staining of parental and the overexpression strain was performed. We noticed that the ectopic expression of VapC21 resulted in the formation of the polar head bulge with condensed nucleoid at 9 h post-Atc induction (data not shown). In contrast, DAPI stained nucleoid was uniformly distributed in vector-only control strain (data not shown). As expected, formation of the polar-head bulge was not observed when both VapC21 and VapB21 were co-expressed in *M. smegmatis* (data not shown).

### Thermal Stability Analysis of the VapBC21 Complex in Comparison to VapB21 and VapC21

For thermal stability assays, VapBC21 complex was purified from the pET-Duet vector, with VapC21 toxin having an amino-terminal 6×-histidine tag. The individually purified VapB21 antitoxin and VapC21 toxin expressed in the pET15b vector both possessed an amino-terminus 6×-histidine tag and carboxy terminus 3×-FLAG tag. The VapBC21 complex and individual proteins were purified using a Ni-NTA purification system. The bound proteins were eluted using an imidazole gradient in the range of 100 mM – 900 mM and analyzed on 15% Tricine-SDS PAGE. The purified fractions were pooled, concentrated and subjected to thermal denaturation. Apparent T_m_ for various proteins at 10 μM concentration was determined by measuring changes in intrinsic fluorescence of tryptophan and tyrosine residues as a function of temperature. As expected, purified VapBC21 complex and VapC21 incubated with 20 μM C-terminal VapB21 peptide showed a much higher T_m_ of 73°C and 62°C, respectively, as compared to the individual VapC21 toxin, which had a T_m_ of 50°C (Figure 1F). In line with published reports, VapB21 was intrinsically disordered and failed to show any proper thermal transition (Figure 1F). These observations indicate that the complexes are more thermally stable in comparison to the individual toxin and antitoxin.

### VapC21 Toxin Is Not Required for Survival of *M. tuberculosis* Under *in vitro* Stress Condition

**FIGURE 2:**
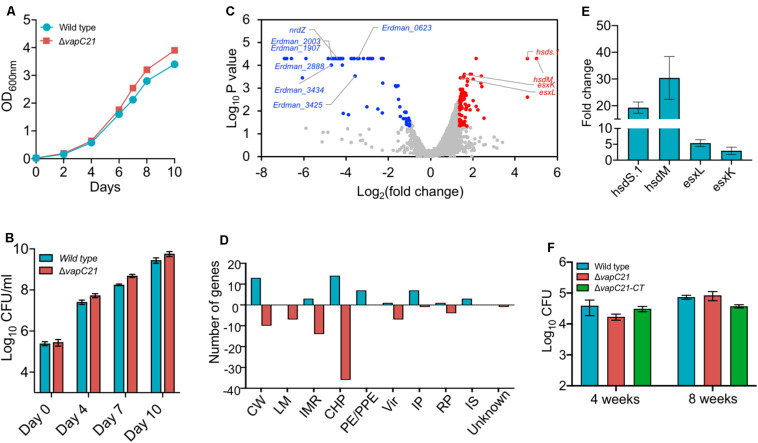
Effect of deletion of VapC21 on the growth, transcriptome and virulence of *M. tuberculosis*. **(A,B)** The growth of the wild type and mutant strain was determined by measuring absorbance OD_600nm_ **(A)** or by CFU enumeration **(B)** until stationary phase. **(C)** The volcano plot showing differential expressed genes between parental and mutant strain. The *Y*-axis and *X*-axis represents log_10_ *P*-value and log_2_ fold change, respectively. The red and blue dots represent DEGs with increased and decreased expression, in the mutant strain, respectively. The transcript levels of genes which remain unchanged between the parental and mutant strain are shown as gray dots. **(D)** The DEGs identified in panel **A** are further categorized according to the functional category mentioned in Mycobrowser. The *Y*-axis represents the number of DEGs for a functional category as mentioned on the *X*-axis. CW, cell wall and cell processes; LM, lipid metabolism; IMR, intermediary metabolism and respiration; CHP, conserved hypothetical protein; PE/PPE, Pro-Glu/Pro-Pro-Glu proteins; Vir, virulence, detoxification, and adaptation; IP, information pathway; RP, regulatory protein; IS, insertion sequences and phages. **(E)** The transcript levels of *hsdS.1*, *hsdM*, *esxL*, *and esxK* was quantified in total RNA isolated from mid-log phase cultures of wild type and Δ*vapC21 M. tuberculosis* strains by qPCR using gene specific primers. The relative expression of these transcripts were obtained after normalization to levels of *sigA*, housekeeping gene. The data shown in this panel is mean ± SE of fold change obtained for each transcript from three independent experiments. **(F)** 6–8 weeks old Female Balb/c mice were infected with either wild type or Δ*vapC21* mutant or Δ*vapC21* complemented strain via the aerosol route. The lung bacillary loads were determined at 4 and 8 weeks post-infection as described in section “Materials and Methods.” The data shown in this panel is mean ± SE of log_10_CFU obtained from 5 animals per group at a given time point. Despite multiple DEGs in the mutant strain, deletion of *vapC21* had no effect on the virulence of *M. tuberculosis*.

To determine the contribution of VapC21 in survival of *M. tuberculosis* under different stress conditions, we constructed a Δ*vapC21* mutant strain of *M. tuberculosis* Erdman using temperature sensitive mycobacteriophages (Bardarov et al., 2002). The replacement of VapC21 open reading frame with the hygromycin resistance gene in the genome of the mutant strain was verified by Southern blot (Supplementary Figures S1A,B and data not shown). As shown in Supplementary Figure S1B, the probe hybridized with 2.0 and 3.7 kb fragments, respectively, in lanes corresponding to *Pvu*II digested genomic DNA from parental and mutant strain, respectively. Both parental and Δ*vapC21* mutant strain displayed identical growth patterns and no defect was observed until late exponential phase in Middlebrook 7H9 medium (Figures 2A,B). In concordance, the deletion of *vapC21* did not alter the colony morphology or biofilm formation of *M. tuberculosis* (Supplementary Figure S1C). Previously, we have shown that the transcript levels of *vapC21* remains unaltered upon exposure to various stress conditions (Agarwal et al., 2018). In agreement, we also observed that the deletion of *vapC21* did not impair the ability of *M. tuberculosis* to survive upon exposure to either oxidative, nitrosative, nutrient starvation, acidic, lysozyme, or detergent stress (Supplementary Figures S1D–G, data not shown). Further, we also compared the survival of parental and mutant strains upon exposure to drugs with different mechanism of action such as isoniazid, levofloxacin, rifampicin or ethambutol. We observed that both strains displayed comparable MIC_99_ values for various drugs evaluated in the study (Supplementary Figure S2A). In agreement, deletion of VapC21 in *M. tuberculosis* genome does not affect persisters formation *in vitro* after exposure to isoniazid, rifampicin or levofloxacin (Supplementary Figure S2B).

### VapC21 Toxin Is Dispensable for *M. tuberculosis* Growth in Mice Model of Infection

In order to gain further mechanistic insights into the role of VapC21 in *M. tuberculosis* physiology, total RNA was isolated from mid-log phase cultures of parental and mutant strains and subjected to RNA-seq analysis. Using a cut-off value of log_2_fold change of 1.0 and *P-value* < 0.05, we observed that approximately 131 genes were differentially expressed between the two strains. Among DEGs, the expression levels of 50 and 81 transcripts were increased or reduced, respectively (Figure 2C and Supplementary Table S2). The observed DEGs were further characterized based on their functional category and the majority of DEGs have been annotated as either conserved hypothetical or cell wall associated proteins (Figure 2D). More detailed analysis of the RNA-seq data revealed that the expression of genes adjacent to VapC21, DNA methyl transferases, *hsds.1* and *hsdM* were increased in the mutant strain (Figure 2C). The transcript levels of ESAT-6 subfamily of small secreted proteins such as *esxK* and *esxL* were increased in the mutant strain in comparison to the parental strain (Figure 2C). The increased expression of *hsds.1*, *hsdM* and *esxK and esxL* in the mutant strain was also validated by qPCR using gene specific primers (Figure 2E). The transcripts of downregulated genes have been annotated as either conserved hypothetical or proteins involved in intermediary metabolism and respiration and cell wall processes. A few of the repressed transcripts such as *nrdZ*, *Erdman_2888*, *Erdman_3434*, *Erdman_1907*, *Erdman_1908*, *Erdman_2003* and *Erdman_3425* belong to the DosR regulon (Park et al., 2003; Chauhan et al., 2011; Boon and Dick, 2012; Supplementary Table S2). Also, the expression of latency associated antigens such as *Erdman_0623*, *Erdman_1907*, *Erdman_1908*, *Erdman_2206* and *otsB1* were also reduced in the mutant strain (Schuck et al., 2009; Supplementary Table S2).

We have earlier reported that strains with deletions in either *vapBC3* or *vapBC4* or *vapBC11* or *vapC22* are attenuated for growth in comparison to the wild type strain in guinea pigs and mice (Agarwal et al., 2018, 2020; Deep et al., 2018). The reduced expression of latency associated genes and genes belonging to the DosR regulon suggests that VapC21 might also be important for *M. tuberculosis* to establish infection in host tissues. In order to investigate the role of VapC21 in *M. tuberculosis* pathogenesis, the growth patterns of wild type, Δ*vapC21* mutant, and Δ*vapC21* complemented strain were compared in a murine model of infection (Figure 2F). The aerosol infection of mice resulted in implantation of approximately 50 bacilli in lungs at day 1 post-infection. As shown in Figure 2F, all three strains displayed comparable growth during both acute (4 weeks) and chronic (8 weeks) stage of infection. The bacterial burdens in lung tissues of mice infected with parental, mutant and complemented strain was approximately log_10_ 4.5 at 8 weeks post-infection (Figure 2F). These findings suggest that VapC21 individually does not contribute to the survival of *M. tuberculosis* in lung tissues.

### Co-expression Studies to Identify Interactions Between VapC21 With Cognate and Non-cognate Antitoxins

**FIGURE 3:**
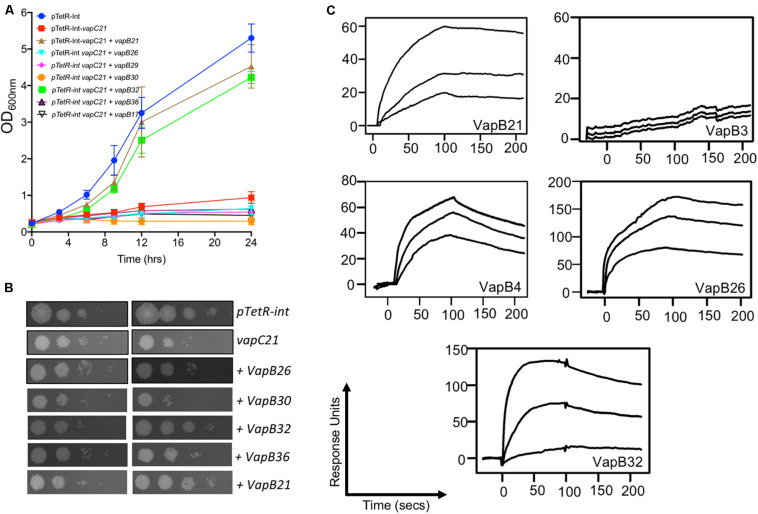
Characterization of interactions of VapC21 with other non-cognate antitoxins by growth inhibition and SPR binding assays. The expression of toxin and antitoxins in early-log phase cultures (OD_600nm_ ∼ 0.2) of recombinant *M. smegmatis* was induced by the addition of 50 ng/ml Atc and 0.2% acetamide, respectively. The growth of various strains was monitored by measuring either OD_600nm_ **(A)** or by spotting assays **(B)** as described in section “Materials and Methods.” The data shown in these panels is representative of two different experiments. **(C)** The binding parameters between cognate and non-cognate TA pairs were determined by SPR as described in section “Materials and Methods.” The kinetic parameters were obtained by fitting the data to the 1:1 Langmuir Interaction model. The overlays show binding kinetics with three different concentration increasing from the bottom to top; 25, 50, and 100 nM in the case of VapB21, 1, 2, and 5 μM in the case of VapB3, 0.5, 1, and 5 μM in the case of VapB4, 0.2, 0.5, and 1 μM in the case of VapB26 and 50, 250, and 500 nM in the case of VapB32.

Under normal physiological conditions the antitoxin levels are in excess of toxins and TA complexes function as auto-repressors (Slayden et al., 2018). Further, in addition to cognate pair, interactions also exist between non-cognate antitoxins and toxins (Zhu et al., 2010; Chen et al., 2019). Next, we performed experiments to determine whether VapC21 is able to interact with other non-cognate antitoxins. The interactions between VapC21 and cognate/non-cognate antitoxins was investigated using growth inhibition and rescue experiments *in vitro*. *M. smegmatis* mc^2^155 harboring an integrative copy of Atc inducible VapC21 was transformed individually with different pLAM12 constructs carrying an acetamide inducible copy of various antitoxins. The expression of toxin and antitoxin in early-log phase cultures of recombinant strains was induced by the addition of Atc and acetamide, respectively. The growth rescue experiments were performed by measuring OD_600nm_ at regular intervals. As expected, minimal growth was observed upon induction of toxin expression in *M. smegmatis* and this was restored upon coexpression of VapC21 along with VapB21 (Supplementary Figure S3A). In our preliminary cross-talk experiments, we observed that co-expression of non-cognate antitoxins VapB17 or VapB26 or VapB29 or VapB30 or VapB32 or VapB36 were able to rescue the growth defect associated with the overexpression of VapC21 (Supplementary Figure S3). However, in our repeat experiments, growth restoration was only observed when VapC21 was co-expressed along with VapB32 (Figure 3A). In agreement, we also observed that VapB32 was able to abrogate the growth inhibition associated with the expression of VapC21 in our spotting assays (Figure 3B). These experiments suggest that in addition to VapB21, VapC21 might be able to interact with non-cognate antitoxin, VapB32.

### Analysis of the Interaction Between VapB21 With Cognate and Non-cognate Antitoxins Using Biophysical Methods

Next, we determined the strength of interaction of binding between VapC21 with purified cognate and non-cognate VapB antitoxins (VapB3, VapB4, VapB26, and VapB32) using SPR (Figure 3C). These antitoxins were selected as in our growth inhibition studies, co-expression of VapB3, VapB4, and VapB26 had no effect where as VapB32 overexpression was able to abrogate the growth inhibition activity of VapC21. The non-cognate antitoxins were purified as (His)_6_-tagged proteins using Ni-NTA based affinity chromatography. The purified fractions were pooled, concentrated and subjected to SPR analysis. The dissociation constant, K_D_ was calculated for each set of interaction as shown in Table 1. We observed that VapB21 binds to VapC21 with a K_D_ of ∼ 3 nM. In comparison, non-cognate antitoxins VapB4, VapB26, and VapB32 displayed reduced binding to VapC21. The K_D_ values for binding of VapB4, VapB26 and VapB32 with VapC21 was 70, 53, and 18 nM, respectively (Figure 3C and Table 1). In the case of VapB4, we observed ∼ 10.0-fold decrease in both association and dissociation rates in comparison to VapB21. Also, we observed ∼ 10.0 fold decrease in the association and ∼ 10.0 fold decrease in the dissociation rate in the case of VapB26 and VapB32, respectively, with VapC21 (Figure 3C and Table 1). In agreement with co-expression data, we did not observe any binding between VapB3 and VapC21. These observations suggest that VapC21 is able to interact with non-cognate VapB antitoxins, in particular VapB32 *in vitro*.

**TABLE 1:** Kinetic parameters of binding of VapC21 with cognate and non-cognate VapB antitoxins.

Proteins	ka (M^–1^s^–1^)	kd (s^–1^)	K_D_ (nM)
VapB21	4.1 ± 1.9 × 10^5^	3.8 ± 0.3 × 10^–4^	3 ± 3
VapB3^a^	–	–	–
VapB4	3.9 ± 2.0 × 10^4^	1.4 ± 0.2 × 10^–3^	73 ± 10
VapB26	1.6 ± 1.0 × 10^4^	6.9 ± 0.8 × 10^–4^	53 ± 7
VapB32	1.3 ± 0.6 × 10^5^	2.0 ± 0.8 × 10^–3^	18 ± 6

*The data shown in this panel is mean ± SE of values obtained from two independent experiments performed in duplicates. ^a^No binding with VapC21 was observed.*

### SEC-MALS Analysis to Determine the Oligomeric States of Various Proteins

**FIGURE 4:**
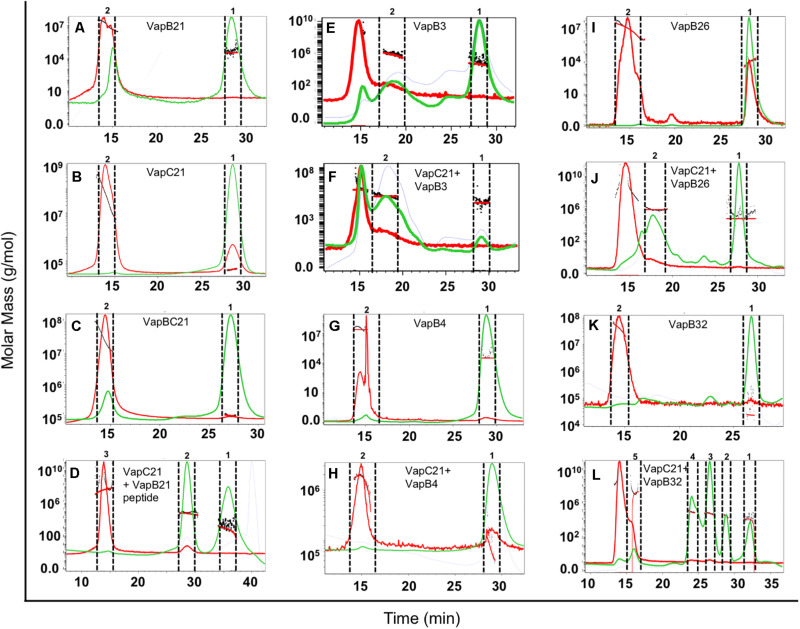
SEC-MALS analysis to determine oligomeric states of the purified proteins. The traces for UV, refractive index and light scattering are shown in green, blue, and red, respectively. The molar mass of all traces are plotted as a function of elution time. The different panels in the figure represent **(A)** VapB21, **(B)** VapC21, **(C)** VapBC21, **(D)** VapC21 with excess VapB21 C-ter peptide, **(E)** VapB3, **(F)** VapC21 with excess VapB3, **(G)** VapB4, **(H)** VapC21 with excess VapB4, **(I)** VapB26, **(J)** VapC21 with excess VapB26, **(K)** VapB32, and **(L)** VapC21 with excess VapB32. The data shown in this panel is representative of two different experiments.

The oligomeric states of different purified proteins were analyzed by SEC-MALS under non-denaturing conditions. We observed two to three different peaks in the UV trace for VapBC21, VapB21-Cterminal peptide-VapC21, VapB3-VapC21, VapB4-VapC21, VapB26-VapC21 and VapB32-VapC21 (Figure 4). The molecular weights of various peaks were calculated and are shown in Table 2. The peak 1 of VapBC21 constitutes 87% of the total fraction and the molecular weight of 103 kDa corresponds to the hetero-octameric complex of T_4_A_4_ (Figure 4C). Further, the peak 2 obtained upon incubation of VapC21 with excess of C-terminal VapB21 peptide constitutes 51% of the total fraction (Figure 4D). The peak corresponded to the hetero-tetrameric form of complex, T_2_A_2_ with a molecular weight of 51 kDa (Figure 4D). However, in the *in vitro* formed complexes of VapB4 and VapB26 with VapC21, the major peak constitutes 89% and 49%, with a molecular weight of 67 kDa and 51 kDa, respectively (Figures 4H,J). These peaks corresponds to hetero-tetrameric form of the complex, T_2_A_2_. As shown in Figure 4L and Table 2, the peak 2 and peak 3 obtained upon incubation of excess VapB32 with VapC21, constitutes 33% and 27% of the total fraction and represents the molecular weight of 60 kDa and 94 kDa, respectively. The peak 2 and peak 3 corresponds to hetero-tetrameric (T_2_A_2_) and hetero-octameric form of the complex (T_4_A_4_), respectively (Figure 4L and Table 2). The obtained peak 1 in the case of VapB3, VapB4, VapB21, VapB26, VapB32 and VapC21 represents their dimeric form with an approximate molecular weight of 31, 30, 32, 19, 23, and 39.2 kDa, respectively (Figures 4A,B,E,G,I,K). However, the exact oligomeric status of complexes obtained with SEC-MALS, can differ from what is observed *in vivo*, owing to the small size of the individual toxins, antitoxins and inability of the column to resolve such small differences in molecular weight.

**TABLE 2:** The mass fractions and corresponding molecular weights of each peak for the proteins analyzed are represented (T, toxin, A, antitoxin).

Proteins	Peak	Molecular weight (kDa)	Mass fraction (%)	Stoichiometry
VapC21	1 2	39 4479	98 2	T_2_ (Dimer) Aggregate
VapB21	1 2	32 1809	66 34	A_2_ (Dimer) Aggregate
VapBC21	1 2	103 2585	77 23	T_4_A_4_ (Hetero-Octamer) Aggregate
VapC21 + excess VapB21 peptide	1 2 3	8 51 5552	48 51 1	Free Peptide AT-TA (Hetero-Tetramer) Aggregate
VapB3	1 2	31 3046	53 41	A_2_ (Dimer) Aggregate
VapC21 + excess VapB3	1 2	39 3446	23 41	A_2_ (Dimer) Aggregate
VapB4	1 2	30 2378	98 2	A_2_ (Dimer) Aggregate
VapC21 + excess VapB4	1 2	67 1601	84 16	T_2_A_2_ (Hetero-Tetramer) Aggregate
VapB26	1 2	19 1829	99 1	A_2_ (Dimer) Aggregate
VapC21 + excess VapB26	1 2	51 6894	49 51	T_2_A_2_ (Hetero-Tetramer) Aggregate
VapB32	1 2	23 4249	86 14	A_2_ (Dimer) Aggregate
VapC21 + excess VapB32	1 2 3 4 5	11 23 60 94 2891	18 15 33 27 7	Degraded Antitoxin Free Antitoxin A_2_ (Dimer) T_2_A_2_ (Hetero-Tetramer) T_4_A_4_ (Hetero-Octamer) Aggregate

### Transcriptional Response to VapC21 Overexpression in *M. tuberculosis*

**FIGURE 5:**
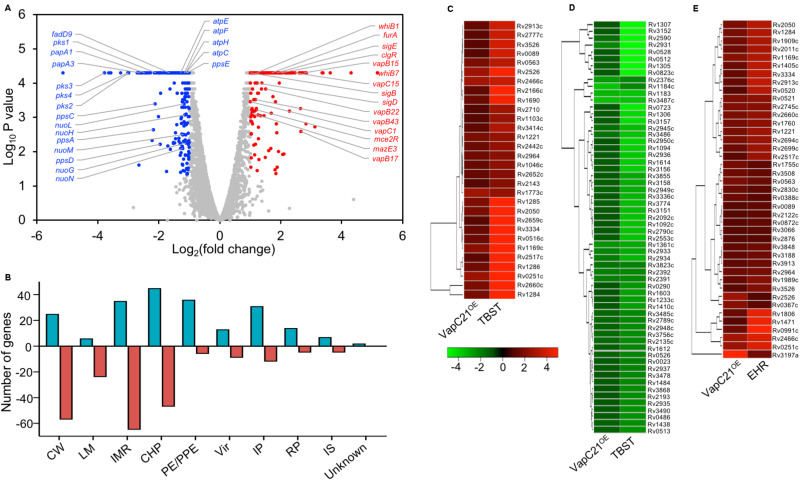
Effect of overexpression of VapC21 on the transcriptome of *M. tuberculosis*. **(A)** The overexpression of VapC21 resulted in differential expression profiles of 445 transcripts. The transcripts whose expression were either increased (red dots) or decreased (blue dots) or remained unchanged (gray dots) are shown in the Volcano plot. The *Y*-axis and *X*-axis represents log_10_ *P*-value and log_2_ fold change, respectively. **(B)** The DEGs identified in panel A have been categorized as per the functional category mentioned in Mycobrowser. The different functional categories have been described in legend to Figure 2D. The *Y*-axis represents the number of DEGs for a given functional category and these are mentioned on *X*-axis. **(C–E)** Heat maps showing fold change of common DEGs that are upregulated **(C)** and downregulated **(D)** in VapC21 overexpression strain and nutritionally starved bacteria. The panel **(E)** shows DEGs that are upregulated both during enduring hypoxic response and upon VapC21 overexpression.

**FIGURE 6:**
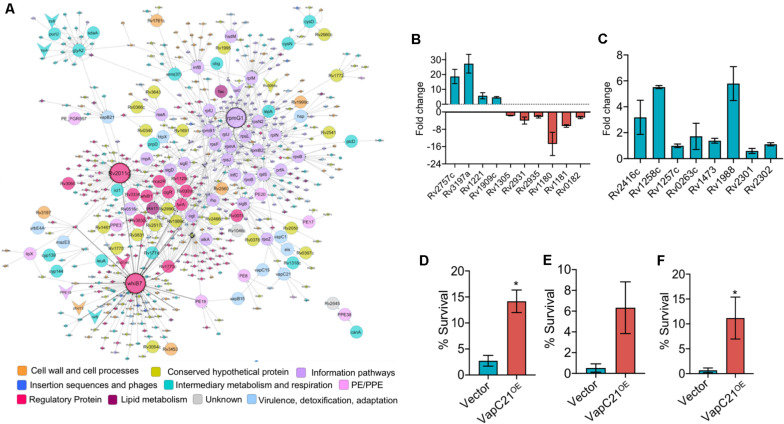
Overexpression of VapC21 confers increased drug tolerance in the presence of amikacin and ethambutol. **(A)** Top-response network of VapC21 overexpression strain compared to wildtype. Nodes are colored based upon Mycobrowser functional categories. The interactions/edges between the nodes are represented by gray arrows. The shape of the nodes represents the pattern of gene expression, circle: induced/upregulated, arrowhead: repressed/downregulated and the non-differentially expressed genes are represented as diamonds. Top hub nodes (nodes with multiple interactions in the top-response network) whiB7, rpmG1, and Rv2011c are highlighted with the black border. **(B,C)** The transcript levels of DEGs **(B)** and WhiB7 regulon genes **(C)** were quantified in early-log phase cultures of parental and VapC21 overexpression *M. tuberculosis* strain by qPCR using gene specific primers. The relative expression of these transcripts was obtained after normalization to levels of *sigA*, housekeeping gene. The data shown in this panel is mean ± SE of fold change obtained for each transcript from two independent experiments. **(D–F)** The expression of toxin was induced in *M. smegmatis* by the addition of 50 ng/ml Atc for 12 h. The cultures were diluted and exposed to medium containing either amikacin **(D)** or streptomycin **(E)** or ethambutol **(F)** for 12 h. The bacterial enumeration and percentage survival was calculated as described in section “Materials and Methods.” The results shown are mean ± SE of percentage survival obtained from three or four independent experiments. Statistically significant differences were obtained for the indicated groups (Paired two-tailed *t*-test, *P < *0.05*).

Several studies have shown that overexpression of toxins belonging to TA systems result in transcriptional reprogramming that might enable the bacteria to adapt to different stress conditions (Singh et al., 2010; Deep et al., 2018). We next performed RNA-seq analysis to compare the transcription profiles of parental and VapC21 overexpression strain. For transcription profiling, total RNA was isolated from early-log phase cultures of various strains and subjected to RNA-seq analysis. Using a cut-off of log_2_ fold change of ≥1.0 or ≤-1.0 and *P-value* < 0.05, we observed that the overexpression of VapC21 in *M. tuberculosis* altered the expression of 445 genes (Figure 5A and Supplementary Table S3). Among these differentially expressed genes, 215 and 230 transcripts were either upregulated or downregulated, respectively (Figure 5A and Supplementary Table S3). These DEGs were further annotated according to their functional category as shown in Figure 5B. Among the DEGs with reduced expression, approximately 29% and 23% of the proteins are involved in intermediary metabolism and respiration and cell wall processes, respectively (Figure 5B). The expression of enzymes involved in lipid metabolism of *M. tuberculosis* such as *pks1*, *pks2*, *pks3, pks4, papA3, papA1, ppsA, ppsC, ppsD, ppsE, mmpL8, fadD9, eccCb1, fadE21, eccCa1, eccB1, fadD22, echA21, fadE18, echA1, eccD3*, and *eccA1* were significantly reduced in the overexpression strain (Supplementary Table S3). The expression of enzymes belonging to either ATP or NADH biosynthesis such as *atpE*, *atpF*, *atpH*, *atpC*, *nuoN*, *nuoM*, *nuoL*, *nuoH*, and *nuoG* were also decreased in the overexpression strain. The transcript levels of *esxK*, *esxL* and *hsdS.1* which were increased in the mutant strain were observed to be decreased in the overexpression strain. In agreement with earlier reports, transcript levels of non-cognate toxins and antitoxins such as *vapC1*, *mazE3*, *vapB43*, *vapB22*, *vapC15*, *vapB17, Rv0366c*, and *vapB15* were also increased in the VapC21 overexpression strain (Supplementary Table S3; Agarwal et al., 2018; Deep et al., 2018). DEGs annotated as regulatory proteins such as *sigB, mce2R, whiB1, sigD, furA, whiB7, sigE, clgR* were also upregulated in the overexpression strain (Supplementary Table S3). We also observed that the transcriptional response obtained upon VapC21 overexpression shared considerable overlap with the bacterial responses upon exposure to different stress conditions such as in nutrient starvation and enduring hypoxic response (Figures 5C–E). Among the identified DEGs, 90 and 41 transcripts were also differentially expressed in *M. tuberculosis* during nutrient starvation and enduring hypoxic response, respectively (Figures 5C–E; Betts et al., 2002; Rustad et al., 2008). The expression profile of a subset of DEGs in the VapC21 overexpression strain was confirmed by qPCR using gene specific primers (Figure 6B).

### *M. tuberculosis* VapC21 Toxin Contribute to Persister Formation *in vitro*

Further, we performed network based analysis on DEGs observed between parental and VapC21 overexpression strain. The master protein-protein interaction network of *M. tuberculosis*, consists of 3,686 proteins (nodes) and 34,223 molecular interactions (edges) (Mishra et al., 2017). The subnetwork formed by the top-ranked paths consisted of 567 nodes (and 901 edges), of which 113 DEGs were identified in our overexpression RNA-seq data (Figure 6A). Among these, the expression of 105 and 8 transcripts were increased and decreased, respectively. The subnetwork was analyzed to identify hub nodes (nodes having higher number of interactions with other nodes), of which *whib7* (regulatory protein) and *rpmG1* (ribosomal protein) were identified as the highest degree hubs. Further, other proteins belonging to functional category of regulatory protein or information pathways such as *sigB*, *sigE*, *rplS*, *rplJ*, *rplM*, and *rpsN2* were also identified as hub-nodes in our networks (Figure 6A). In agreement, the transcript levels of genes that are known to be regulated by WhiB7, such as *eis* (Rv2416c, aminoglycoside acetyltransferase) and Rv1258c (efflux pump) and Rv1988 were also increased in the overexpression strain (Figure 6C). However, the expression levels of other transcripts for Rv0263c, Rv1257c, Rv1473, Rv2301, and Rv2302 belonging to WhiB7 regulon was comparable in the parental and overexpression strain (Figure 6C). Previously it has been reported that the expression of *whib7* is induced upon response to aminoglycosides, stress conditions and is also associated with intrinsic drug-resistance mechanisms (Morris et al., 2005; Burian et al., 2012; Reeves et al., 2013). This prompted us to investigate whether VapC21 contributes to the formation of drug-tolerant persisters in mycobacteria upon exposure to aminoglycosides. We observed that VapC21 overexpression in *M. smegmatis* increased the number of amikacin tolerant and streptomycin tolerant persisters by 5.0-fold and 12.0-fold, respectively (Figures 6D,E, *P < *0.05*). In addition to WhiB7, the transcript levels of isoniazid inducible operon, *iniBAC* (Rv0340c-Rv0342c) were also increased in VapC21 overexpression strain. Studies have shown that increased levels of *iniBAC* operon is associated with the emergence of isoniazid and ethambutol tolerance (Alland et al., 2000; Colangeli et al., 2005). Therefore, we also determined the effect of VapC21 overexpression on emergence of ethambutol tolerant persisters in *M. smegmatis*. As shown in Figure 6F, overexpression of VapC21 also conferred an increase in the frequency of ethambutol tolerant persisters by 16.0-fold (*P < *0.05*). Taken together, these observations suggest that overexpression of VapC21 increased the percentage of bacterial population that survived killing upon exposure to both aminoglycosides and ethambutol.

## Discussion

The complex molecular mechanisms of regulatory networks that coordinate mycobacterial adaptation during infection culminating into an active or latent disease and/or subsequent reactivation are poorly understood. *M. tuberculosis* deploys a multitude of regulatory networks to survive under different stress conditions in host tissues. The repertoire of TA systems is highly conserved in members belonging to the *M. tuberculosis* complex and a few of these have been biochemically and functionally characterized (Ramage et al., 2009; Tandon et al., 2019b). In the present study, we have functionally and biochemically characterized the VapBC21 TA system from *M. tuberculosis*. In concordance with previous reports, we report that overexpression of VapC21 inhibited growth of *M. smegmatis* in a bacteriostatic manner (Tiwari et al., 2015; Agarwal et al., 2018). The observed growth inhibition upon overexpression of VapC21 could be restored upon coexpression of cognate antitoxin. Live-dead imaging revealed that *M. bovis* BCG overexpressing VapC21 were viable as reported in the case of overexpression of MazF, RelE, VapCs and ParE toxins. Previous studies have shown that expression of toxins belonging to TA systems results in morphological changes, such as formation of lemon shaped cells (Masuda et al., 2012; Wang et al., 2012). We also observed that overexpression of VapC21 resulted in bulge formation in *M. smegmatis* while no changes were observed in strains harboring the vector control. Also, in concordance with previous studies, we observed that nucleoid was more localized toward the poles in VapC21 overexpression strain. We hypothesized that morphological changes observed upon VapC21 overexpression could be attributed to the reduced expression of enzymes involved in either peptidoglycan biosynthesis or cell wall synthesis or cell division proteins.

In order to establish a successful infection, *M. tuberculosis* adapts to a variety of stress conditions that it encounters in the host. Several studies have shown that subsets of TA systems are differentially expressed upon exposure of *M. tuberculosis* to stress conditions such as oxidative or low oxygen or nutrient limiting conditions (Ramage et al., 2009; Agarwal et al., 2018). These observations indicates that in addition to their postulated role in genome stability, TA systems might work synergistically to enable bacteria to adapt to different stress conditions and persist in host tissues. In order to understand the role of VapC21 in *M. tuberculosis* physiology, a Δ*vapC21* mutant strain was generated using temperature sensitive mycobacteriophages. In agreement, with previously published gene essentiality data, we demonstrate that VapC21 is dispensable for survival of *M. tuberculosis in vitro* in different conditions. TA systems have been implicated to regulate pathogenesis in *Uropathogenic E. coli*, *S. typhimurium*, *H. influenzae* and *M. tuberculosis* (Norton and Mulvey, 2012; Ren et al., 2012; De la Cruz et al., 2013; Tiwari et al., 2015; Agarwal et al., 2018; Deep et al., 2018). Previously, we have shown that both MazF and VapC ribonucleases contribute to the ability of *M. tuberculosis* to establish infection in host tissues. These studies suggested that TA systems such as MazF3, MazF6, MazF9, VapBC3, VapBC4, VapBC11, and VapC22 enable *M. tuberculosis* to adapt to changes associated with the onset of host adaptive immunity (Tiwari et al., 2015; Agarwal et al., 2018, 2020; Deep et al., 2018). Despite the reduced expression of genes belonging to the dormancy regulon or latency associated antigens in the mutant strain, we did not observe any significant differences in the lung bacillary loads in mice infected with various strains until 8 weeks post-infection. Taken together, these findings indicate that similar to VapC28, RelE1, RelE2, RelE3 and DarTG, VapC21 is also dispensable for *M. tuberculosis* growth in liquid cultures, stress adaptation and in host tissues (Singh et al., 2010; Agarwal et al., 2018; Zaveri et al., 2020).

Several studies have shown that TA systems constitute an interactome instead of the one-to-one interaction model (Zhu et al., 2010; Chen et al., 2019). For example, Zhu et al. (2010) showed that Rv1991c in addition to Rv1991a also interacts with Rv2801a, Rv0599c, and Rv2595. Also, recently, it has been reported that Rv2063a interacts with both Rv2063 and Rv2801a (Chen et al., 2019). However, the strength of interactions between members of a cognate TA pair was relatively stronger in comparison to those observed in non-cognate TA pairs (Chen et al., 2019). In the present study, we observed that in addition to VapB21, co-expression of VapB32 was able to abrogate growth inhibition associated with overexpression of VapC21 in *M. smegmatis*. SPR studies revealed the strongest binding of VapC21 was observed with VapB21 followed by non-cognate antitoxins, VapB32, VapB26, VapB4 and VapB3. In agreement, with the reduced binding affinity of VapB26, VapB4 and VapB3 with VapC21 we did not observe any growth restoration upon co-expression of these antitoxins. SEC-MALS analysis revealed that complexes formed between VapC21 and antitoxins VapB21 (cognate) and VapB32 (non-cognate) were hetero-octamers, whereas hetero-tetrameric complexes were formed between VapC21 and non-cognate antitoxins VapB4 and VapB26. Based on these observations, we speculate that TA systems indeed constitute a regulatory network that enables *M. tuberculosis* to restore growth from stressful conditions. Since these non-cognate TA pair interactions are relatively weaker in strength in comparison to the cognate TA pair, we hypothesize that under cellular conditions with reduced levels of cognate antitoxin, these heterologous interactions could facilitate fine tuning the activity of non-cognate toxins *in vivo*.

The analysis of the transcriptional response to the ectopic expression of VapC21 in *M. tuberculosis* revealed that the presence of free toxin results in transcriptional reprogramming that overlapped substantially with responses observed upon exposure of bacteria to different stress conditions. The observed pleiotropic response resulting in differential gene expression of 445 transcripts could be attributed to increased transcript levels of regulatory proteins and other non-cognate toxins. Network analysis identified the WhiB7 regulon among the top-response network upon overexpression of VapC21. WhiB7 has been previously reported to be upregulated in response to various antibiotics and is associated with intrinsic drug resistance (Morris et al., 2005; Burian et al., 2012; Reeves et al., 2013). In addition to WhiB7 the transcript levels of another antibiotic response operon, *iniBAC*, which activates and provides the defense against cell wall inhibiting antibiotics isoniazid and ethambutol were also increased in the overexpression strain (Alland et al., 2000; Colangeli et al., 2005). In agreement, we also observed that overexpression of VapC21 led to an increase in amikacin-tolerant and ethambutol-tolerant persisters in *M. smegmatis*. Although, the contribution of Type II TA systems in bacterial persistence and drug tolerance are controversial, our results are in agreement with other studies, where overexpression of Type II toxins is associated with increased tolerance of *E. coli*, *M. smegmatis* or *M. tuberculosis* upon exposure to various drugs (Keren et al., 2004; Dorr et al., 2010; Singh et al., 2010; Holden and Errington, 2018; Ronneau and Helaine, 2019; Tandon et al., 2019a).

In summary, we have functionally characterized the VapBC21 TA system from *M. tuberculosis*. We report that in addition to cognate antitoxin, VapC21 is also able to interact with the non-cognate antitoxin, VapB32. We speculate that cross-talk between non-cognate TA pairs results in a complex regulatory network that fine tunes the activity of toxin and might enable the bacteria to reactivate from a dormant state. VapC21 is non-essential for *in vivo* growth but is involved in the generation of amikacin-tolerant and ethambutol-tolerant persisters *in vitro*. These observations suggests that mycobacterial ribonucleases belong to a regulatory network that contributes to disease reactivation and relapse.

## Data Availability Statement

The RNA-seq data discussed in the present study has been deposited in NCBI’s Gene Expression Omnibus and are accessible through GEO Series accession number GSE154320.

## Ethics Statement

The animal study was reviewed and approved by Translational Health Science and Technology Institute Animal Ethics Committee.

## Author Contributions

RS conceived the idea and designed the experiments. AS performed *M. tuberculosis* microbiology experiments. SAg and AS performed mice experiments. PC and AS performed growth rescue experiments. GC and SAh performed protein purification and biophysical characterization studies. MB and CT helped with the analysis of RNA-seq data. RS, RV, and NC supervised the experiments. RS, GC, and AS wrote the manuscript with inputs from other authors. All authors contributed to the article and approved the submitted version.

## Conflict of Interest

The authors declare that the research was conducted in the absence of any commercial or financial relationships that could be construed as a potential conflict of interest.

## Footnotes

1 http://www.networkdynamics.org/static/zen/html/api/algorithms/shortest_path.html

## Supplementary Material

FIGURE S1 Deletion of *vapC21* does not affect growth of *M. tuberculosis* in different conditions. **(A,B)** Schematic representation for the construction of Δ*vapC21* strain *of M. tuberculosis* Erdman. **(A)** The open reading frame for *vapC21* was replaced with the hygromycin resistance gene in the Δ*vapC21* mutant strain (MT1 and MT2), using temperature sensitive mycobacteriophages. **(B)** The replacement of *vapC21* with the hygromycin resistance gene in the mutant strain was confirmed by Southern blot. Solid line (P) depicts the region for hybridization of probes. WT represent wild type strain of *M. tuberculosis*. **(C)** The colony morphology and biofilm formation of the wild type and Δ*vapC21* mutant strain was determined as described in section “Materials and Methods.” **(D–G)** For stress experiments, early-log phase cultures (OD_600nm_ ∼ 0.2) were exposed to different stress conditions such as oxidative stress **(D)**, nitrosative stress **(E)**, nutrient starvation **(F)**, and 2.5 mg/ml lysozyme **(G)** as described in section “Materials and Methods.” The data shown in panels **(D–G)** are mean ± SE. obtained from triplicate samples and represents two independent experiments.

FIGURE S2 Susceptibility of parental and Δ*vapC21* mutant strain to different drugs *in vitro.* **(A)** This panel shows MIC_99_ values of rifampicin, levofloxacin, isoniazid and ethambutol for early-log phase cultures of parental and Δ*vapC21 M. tuberculosis* strain. **(B)** For drug-tolerance experiment, mid-log phase cultures of various strains were exposed to different drugs for 14 days. For bacterial enumeration, 10.0-fold serial dilutions were prepared and plated on Middlebrook 7H11 at 37°C for 3–4 weeks. The data shown in this panel is mean ± SE obtained from three independent experiments.

FIGURE S3 Co-expression experiments to determine interaction of VapC21 with cognate and non-cognate antitoxins. For co-expression studies, various pLam12-*vapBx* constructs were electroporated into *M. smegmatis* harboring an integrative Atc inducible copy of VapC21. The expression of toxin and antitoxin was induced by the addition of 50 ng/ml Atc and 0.2% acetamide, respectively. The growth of various strains was monitored by measuring OD_600nm_ and data shown in this figure is representative of two independent experiments.

TABLE S1 List of strains and plasmids used in the present study.

TABLE S2 List of transcripts upregulated and downregulated in ΔvapC21 mutant strain in comparison to parental strain by >1.0 fold and *p*-value <0.05.

TABLE S3 List of genes differentially expressed in the overexpression strain in comparison to parental strain with log2 fold change of <1.0 or >1.0 and *P*-value <0.05.

## References

[B1] Agarwal S. Sharma A. Bouzeyen R. Deep A. Sharma H. Mangalaparthi K. K. (2020). VapBC22 toxin-antitoxin system from *Mycobacterium tuberculosis* is required for pathogenesis and modulation of host immune response. *Sci. Adv.* 6:eaba6944. 10.1126/sciadv.aba6944 32537511 PMC7269643

[B2] Agarwal S. Tiwari P. Deep A. Kidwai S. Gupta S. Thakur K. G. (2018). System-wide analysis unravels the differential regulation and in vivo essentiality of virulence-associated Proteins B and C toxin-antitoxin systems of *Mycobacterium tuberculosis*. *J. Infect Dis.* 217 1809–1820. 10.1093/infdis/jiy109 29529224

[B3] Akarsu H. Bordes P. Mansour M. Bigot D. J. Genevaux P. Falquet L. (2019). TASmania: a bacterial toxin-antitoxin systems database. *PLoS Comput. Biol.* 15:e1006946. 10.1371/journal.pcbi.1006946 31022176 PMC6504116

[B4] Alland D. Steyn A. J. Weisbrod T. Aldrich K. (2000). Characterization of the *Mycobacterium tuberculosis* iniBAC promoter, a promoter that responds to cell wall biosynthesis inhibition. *J. Bacteriol.* 182 1802–1811. 10.1128/JB.182.7.1802-1811.2000 10714983 PMC101861

[B5] Arcus V. L. McKenzie J. L. Robson J. Cook G. M. (2011). The PIN-domain ribonucleases and the prokaryotic VapBC toxin-antitoxin array. *Protein Eng. Des. Sel.* 24 33–40. 10.1093/protein/gzq081 21036780

[B6] Arora G. Chaudhary D. Kidwai S. Sharma D. Singh R. (2018). CitE enzymes are essential for *Mycobacterium tuberculosis* to establish infection in macrophages and guinea pigs. *Front. Cell Infect. Microbiol* 8:385. 10.3389/fcimb.2018.00385 30460206 PMC6232273

[B7] Bardarov S. Bardarov S. Jr. Pavelka M. S. Jr. Sambandamurthy V. Larsen M. Tufariello J. (2002). Specialized transduction: an efficient method for generating marked and unmarked targeted gene disruptions in *Mycobacterium tuberculosis*, *M. bovis* BCG and *M. smegmatis*. *Microbiology* 148 3007–3017. 10.1099/00221287-148-10-3007 12368434

[B8] Bendtsen K. L. Brodersen D. E. (2017). Higher-order structure in bacterial vapbc toxin-antitoxin complexes. *Subcell. Biochem.* 83 381–412. 10.1007/978-3-319-46503-6_14 28271484

[B9] Betts J. C. Lukey P. T. Robb L. C. McAdam R. A. Duncan K. (2002). Evaluation of a nutrient starvation model of *Mycobacterium tuberculosis* persistence by gene and protein expression profiling. *Mol. Microbiol.* 43 717–731. 10.1046/j.1365-2958.2002.02779.x 11929527

[B10] Boon C. Dick T. (2012). How *Mycobacterium tuberculosis* goes to sleep: the dormancy survival regulator DosR a decade later. *Future Microbiol.* 7 513–518. 10.2217/fmb.12.14 22439727

[B11] Bruce D. Cardew E. Freitag-Pohl S. Pohl E. (2019). How to stabilize protein: stability screens for thermal shift assays and nano differential scanning fluorimetry in the Virus-X project. *J. Vis. Exp.* e58666. 10.3791/58666 30799847

[B12] Burian J. Ramon-Garcia S. Sweet G. Gomez-Velasco A. Av-Gay Y. Thompson C. J. (2012). The mycobacterial transcriptional regulator whiB7 gene links redox homeostasis and intrinsic antibiotic resistance. *J. Biol. Chem.* 287 299–310. 10.1074/jbc.M111.302588 22069311 PMC3249081

[B13] Chattopadhyay G. Varadarajan R. (2019). Facile measurement of protein stability and folding kinetics using a nano differential scanning fluorimeter. *Protein Sci.* 28 1127–1134. 10.1002/pro.3622 30993730 PMC6511731

[B14] Chauhan S. Sharma D. Singh A. Surolia A. Tyagi J. S. (2011). Comprehensive insights into *Mycobacterium tuberculosis* DevR (DosR) regulon activation switch. *Nucleic Acids Res.* 39 7400–7414. 10.1093/nar/gkr375 21653552 PMC3177182

[B15] Chen R. Tu J. Tan Y. Cai X. Yang C. Deng X. (2019). Structural and biochemical characterization of the cognate and heterologous interactions of the MazEF-mt9 TA system. *ACS Infect. Dis.* 5 1306–1316. 10.1021/acsinfecdis.9b00001 31267737

[B16] Cintron M. Zeng J. M. Barth V. C. Cruz J. W. Husson R. N. Woychik N. A. (2019). Accurate target identification for *Mycobacterium tuberculosis* endoribonuclease toxins requires expression in their native host. *Sci. Rep.* 9:5949. 10.1038/s41598-019-41548-9 30976025 PMC6459853

[B17] Colangeli R. Helb D. Sridharan S. Sun J. Varma-Basil M. Hazbon M. H. (2005). The *Mycobacterium tuberculosis* iniA gene is essential for activity of an efflux pump that confers drug tolerance to both isoniazid and ethambutol. *Mol. Microbiol.* 55 1829–1840. 10.1111/j.1365-2958.2005.04510.x 15752203

[B18] Cook G. M. Robson J. R. Frampton R. A. McKenzie J. Przybilski R. Fineran P. C. (2013). Ribonucleases in bacterial toxin-antitoxin systems. *Biochim. Biophys. Acta* 1829 523–531. 10.1016/j.bbagrm.2013.02.007 23454553

[B19] Cruz J. W. Sharp J. D. Hoffer E. D. Maehigashi T. Vvedenskaya I. O. Konkimalla A. (2015). Growth-regulating *Mycobacterium tuberculosis* VapC-mt4 toxin is an isoacceptor-specific tRNase. *Nat. Commun.* 6:7480. 10.1038/ncomms8480 26158745 PMC4620994

[B20] Das U. Pogenberg V. Subhramanyam U. K. Wilmanns M. Gourinath S. Srinivasan A. (2014). Crystal structure of the VapBC-15 complex from *Mycobacterium tuberculosis* reveals a two-metal ion dependent PIN-domain ribonuclease and a variable mode of toxin-antitoxin assembly. *J. Struct. Biol.* 188 249–258. 10.1016/j.jsb.2014.10.002 25450593

[B21] De la Cruz M. A. Zhao W. Farenc C. Gimenez G. Raoult D. Cambillau C. (2013). A toxin-antitoxin module of *Salmonella* promotes virulence in mice. *PLoS Pathog.* 9:e1003827. 10.1371/journal.pcbi.1003827 24385907 PMC3868539

[B22] Deep A. Kaundal S. Agarwal S. Singh R. Thakur K. G. (2017). Crystal structure of *Mycobacterium tuberculosis* VapC20 toxin and its interactions with cognate antitoxin, VapB20, suggest a model for toxin-antitoxin assembly. *FEBS J.* 284 4066–4082. 10.1111/febs.14289 28986943

[B23] Deep A. Tiwari P. Agarwal S. Kaundal S. Kidwai S. Singh R. (2018). Structural, functional and biological insights into the role of *Mycobacterium tuberculosis* VapBC11 toxin-antitoxin system: targeting a tRNase to tackle mycobacterial adaptation. *Nucleic Acids Res.* 46 11639–11655. 10.1093/nar/gky924 30329074 PMC6265470

[B24] Dorr T. Vulic M. Lewis K. (2010). Ciprofloxacin causes persister formation by inducing the TisB toxin in *Escherichia coli*. *PLoS Biol.* 8:e1000317. 10.1371/journal.pcbi.1000317 20186264 PMC2826370

[B25] Ehrt S. Guo X. V. Hickey C. M. Ryou M. Monteleone M. Riley L. W. (2005). Controlling gene expression in mycobacteria with anhydrotetracycline and Tet repressor. *Nucleic Acids Res.* 33:e21. 10.1093/nar/gni013 15687379 PMC548372

[B26] Ehrt S. Schnappinger D. (2009). Mycobacterial survival strategies in the phagosome: defence against host stresses. *Cell Microbiol.* 11 1170–1178. 10.1111/j.1462-5822.2009.01335.x 19438516 PMC3170014

[B27] Glaziou P. Floyd K. Raviglione M. C. (2018). Global epidemiology of tuberculosis. *Semin. Respir. Crit. Care Med.* 39 271–285. 10.1055/s-0038-1651492 30071543

[B28] Gupta A. (2009). Killing activity and rescue function of genome-wide toxin-antitoxin loci of *Mycobacterium tuberculosis*. *FEMS Microbiol. Lett.* 290 45–53. 10.1111/j.1574-6968.2008.01400.x 19016878

[B29] Harms A. Brodersen D. E. Mitarai N. Gerdes K. (2018). Toxins, targets, and triggers: an overview of toxin-antitoxin biology. *Mol. Cell.* 70 768–784. 10.1016/j.molcel.2018.01.003 29398446

[B30] Holden D. W. Errington J. (2018). Type II Toxin-antitoxin systems and persister cells. *mBio* 9:e005068-11. 10.1128/mBio.01574-18 30254124 PMC6156201

[B31] Jardim P. Santos I. C. Barbosa J. A. de Freitas S. M. Valadares N. F. (2016). Crystal structure of VapC21 from *Mycobacterium tuberculosis* at 1.*31* A resolution. *Biochem. Biophys. Res. Commun.* 478 1370–1375. 10.1016/j.bbrc.2016.08.130 27576202

[B32] Kanehisa M. Goto S. (2000). KEGG: kyoto encyclopedia of genes and genomes. *Nucleic Acids Res.* 28 27–30. 10.1093/nar/28.1.27 10592173 PMC102409

[B33] Kang S. M. Kim D. H. Jin C. Lee B. J. (2018). A systematic overview of Type II and III toxin-antitoxin systems with a focus on druggability. *Toxins* 10:515. 10.3390/toxins10120515 30518070 PMC6315513

[B34] Kang S. M. Kim D. H. Lee K. Y. Park S. J. Yoon H. J. Lee S. J. (2017). Functional details of the *Mycobacterium tuberculosis* VapBC26 toxin-antitoxin system based on a structural study: insights into unique binding and antibiotic peptides. *Nucleic Acids Res.* 45 8564–8580. 10.1093/nar/gkx489 28575388 PMC5737657

[B35] Keren I. Minami S. Rubin E. Lewis K. (2011). Characterization and transcriptome analysis of *Mycobacterium tuberculosis* persisters. *mBio* 2:e00100-11. 10.1128/mBio.00100-11 21673191 PMC3119538

[B36] Keren I. Shah D. Spoering A. Kaldalu N. Lewis K. (2004). Specialized persister cells and the mechanism of multidrug tolerance in *Escherichia coli*. *J. Bacteriol.* 186 8172–8180. 10.1128/JB.186.24.8172-8180.2004 15576765 PMC532439

[B37] Kesavardhana S. Das R. Citron M. Datta R. Ecto L. Srilatha N. S. (2017). Structure-based design of cyclically permuted HIV-1 gp120 trimers that elicit neutralizing antibodies. *J. Biol. Chem.* 292 278–291. 10.1074/jbc.M116.725614 27879316 PMC5217686

[B38] Kidwai S. Park C. Y. Mawatwal S. Tiwari P. Jung M. G. Gosain T. P. (2017). Dual Mechanism of action of 5-Nitro-1,10-Phenanthroline against *Mycobacterium tuberculosis*. *Antimicrob. Agents Chemother.* 61:e00969-17. 10.1128/AAC.00969-17 28893784 PMC5655107

[B39] Kim D. Pertea G. Trapnell C. Pimentel H. Kelley R. Salzberg S. L. (2013). TopHat2: accurate alignment of transcriptomes in the presence of insertions, deletions and gene fusions. *Genome Biol.* 14:R36. 10.1186/gb-2013-14-4-r36 23618408 PMC4053844

[B40] Langmead B. Salzberg S. L. (2012). Fast gapped-read alignment with Bowtie 2. *Nat. Methods* 9 357–359. 10.1038/nmeth.1923 22388286 PMC3322381

[B41] Lee I. G. Lee S. J. Chae S. Lee K. Y. Kim J. H. Lee B. J. (2015). Structural and functional studies of the *Mycobacterium tuberculosis* VapBC30 toxin-antitoxin system: implications for the design of novel antimicrobial peptides. *Nucleic Acids Res.* 43 7624–7637. 10.1093/nar/gkv689 26150422 PMC4551927

[B42] Li H. Handsaker B. Wysoker A. Fennell T. Ruan J. Homer N. (2009). The sequence alignment/Map format and SAMtools. *Bioinformatics* 25 2078–2079. 10.1093/bioinformatics/btp352 19505943 PMC2723002

[B43] Lobato-Marquez D. Diaz-Orejas R. Garcia-Del Portillo F. (2016). Toxin-antitoxins and bacterial virulence. *FEMS Microbiol. Rev.* 40 592–609. 10.1093/femsre/fuw022 27476076

[B44] Magnusson A. O. Szekrenyi A. Joosten H. J. Finnigan J. Charnock S. Fessner W. D. (2019). nanoDSF as screening tool for enzyme libraries and biotechnology development. *FEBS J.* 286 184–204. 10.1111/febs.14696 30414312 PMC7379660

[B45] Masuda H. Tan Q. Awano N. Wu K. P. Inouye M. (2012). YeeU enhances the bundling of cytoskeletal polymers of MreB and FtsZ, antagonizing the CbtA (YeeV) toxicity in *Escherichia coli*. *Mol. Microbiol.* 84 979–989. 10.1111/j.1365-2958.2012.08068.x 22515815

[B46] Miallau L. Faller M. Chiang J. Arbing M. Guo F. Cascio D. (2009). Structure and proposed activity of a member of the VapBC family of toxin-antitoxin systems, *VapBC-*5 from *Mycobacterium tuberculosis*. *J. Biol. Chem.* 284 276–283. 10.1074/jbc.M805061200 18952600 PMC2610494

[B47] Min A. B. Miallau L. Sawaya M. R. Habel J. Cascio D. Eisenberg D. (2012). The crystal structure of the Rv0301-Rv0300 VapBC-3 toxin-antitoxin complex from *M. tuberculosis* reveals a *Mg(*2)(+) ion in the active site and a putative RNA-binding site. *Protein Sci.* 21 1754–1767. 10.1002/pro.2161 23011806 PMC3527712

[B48] Mishra S. Shukla P. Bhaskar A. Anand K. Baloni P. Jha R. K. (2017). Efficacy of beta-lactam/beta-lactamase inhibitor combination is linked to WhiB4-mediated changes in redox physiology of *Mycobacterium tuberculosis*. *eLife* 6:e25624 10.7554/eLife.25624.037 PMC5473688 28548640

[B49] Morris R. P. Nguyen L. Gatfield J. Visconti K. Nguyen K. Schnappinger D. (2005). Ancestral antibiotic resistance in *Mycobacterium tuberculosis*. *Proc. Natl. Acad. Sci. U.S.A.* 102 12200–12205. 10.1073/pnas.0505446102 16103351 PMC1186028

[B50] Muthuramalingam M. White J. C. Bourne C. R. (2016). Toxin-antitoxin modules are pliable switches activated by multiple protease pathways. *Toxins* 8:214. 10.3390/toxins8070214 27409636 PMC4963847

[B51] Norton J. P. Mulvey M. A. (2012). Toxin-antitoxin systems are important for niche-specific colonization and stress resistance of uropathogenic *Escherichia coli*. *PLoS Pathog.* 8:e1002954. 10.1371/journal.pcbi.1002959 23055930 PMC3464220

[B52] Page R. Peti W. (2016). Toxin-antitoxin systems in bacterial growth arrest and persistence. *Nat. Chem. Biol.* 12 208–214. 10.1038/nchembio.2044 26991085

[B53] Pandey D. P. Gerdes K. (2005). Toxin-antitoxin loci are highly abundant in free-living but lost from host-associated prokaryotes. *Nucleic Acids Res.* 33 966–976. 10.1093/nar/gki201 15718296 PMC549392

[B54] Park H. D. Guinn K. M. Harrell M. I. Liao R. Voskuil M. I. Tompa M. (2003). Rv3133c/dosR is a transcription factor that mediates the hypoxic response of *Mycobacterium tuberculosis*. *Mol. Microbiol.* 48 833–843. 10.1046/j.1365-2958.2003.03474.x 12694625 PMC1992516

[B55] Ramage H. R. Connolly L. E. Cox J. S. (2009). Comprehensive functional analysis of *Mycobacterium tuberculosis* toxin-antitoxin systems: implications for pathogenesis, stress responses, and evolution. *PLoS Genet.* 5:e1000767 10.1371/journal.pcbi.100767 PMC2781298 20011113

[B56] Reeves A. Z. Campbell P. J. Sultana R. Malik S. Murray M. Plikaytis B. B. (2013). Aminoglycoside cross-resistance in *Mycobacterium tuberculosis* due to mutations in the 5′ untranslated region of whiB7. *Antimicrob. Agents Chemother.* 57 1857–1865. 10.1128/AAC.02191-12 23380727 PMC3623337

[B57] Ren D. Walker A. N. Daines D. A. (2012). Toxin-antitoxin loci vapBC-1 and vapXD contribute to survival and virulence in nontypeable *Haemophilus influenzae*. *BMC Microbiol.* 12:263 10.1186/s12864-016-2792-263 PMC3560280 23157645

[B58] Ronneau S. Helaine S. (2019). Clarifying the Link between Toxin-antitoxin modules and bacterial persistence. *J. Mol. Biol.* 431 3462–3471. 10.1016/j.jmb.2019.03.019 30914294

[B59] Rustad T. R. Harrell M. I. Liao R. Sherman D. R. (2008). The enduring hypoxic response of *Mycobacterium tuberculosis*. *PLoS One* 3:e1502 10.1371/journal.pcbi.01502 PMC2198943 18231589

[B60] Sambarey A. Prashanthi K. Chandra N. (2013). Mining large-scale response networks reveals ‘topmost activities’ in *Mycobacterium tuberculosis* infection. *Sci. Rep.* 3:2302. 10.1038/srep02302 23892477 PMC3725478

[B61] Sambaturu N. Mishra M. Chandra N. (2016). EpiTracer – an algorithm for identifying epicenters in condition-specific biological networks. *BMC Genomics* 17(Suppl. 4):543. 10.1186/s12864-016-2792-1 27556637 PMC5001201

[B62] Schuck S. D. Mueller H. Kunitz F. Neher A. Hoffmann H. Franken K. L. (2009). Identification of T-cell antigens specific for latent *mycobacterium tuberculosis* infection. *PLoS One* 4:e5590 10.1371/journal.pcbi.05590 PMC2680040 19440342

[B63] Schuster C. F. Bertram R. (2013). Toxin-antitoxin systems are ubiquitous and versatile modulators of prokaryotic cell fate. *FEMS Microbiol. Lett.* 340 73–85. 10.1111/1574-6968.12074 23289536

[B64] Shannon P. Markiel A. Ozier O. Baliga N. S. Wang J. T. Ramage D. (2003). Cytoscape: a software environment for integrated models of biomolecular interaction networks. *Genome Res.* 13 2498–2504. 10.1101/gr.1239303 14597658 PMC403769

[B65] Sharp J. D. Cruz J. W. Raman S. Inouye M. Husson R. N. Woychik N. A. (2012). Growth and translation inhibition through sequence-specific RNA binding by *Mycobacterium tuberculosis* VapC toxin. *J. Biol. Chem.* 287 12835–12847. 10.1074/jbc.M112.340109 22354968 PMC3339977

[B66] Singh R. Barry C. E. III Boshoff H. I. (2010). The three RelE homologs of *Mycobacterium tuberculosis* have individual, drug-specific effects on bacterial antibiotic tolerance. *J. Bacteriol.* 192 1279–1291. 10.1128/JB.01285-09 20061486 PMC2820853

[B67] Singh R. Singh M. Arora G. Kumar S. Tiwari P. Kidwai S. (2013). Polyphosphate deficiency in *Mycobacterium tuberculosis* is associated with enhanced drug susceptibility and impaired growth in guinea pigs. *J. Bacteriol.* 195 2839–2851. 10.1128/JB.00038-13 23585537 PMC3697247

[B68] Slayden R. A. Dawson C. C. Cummings J. E. (2018). Toxin-antitoxin systems and regulatory mechanisms in *Mycobacterium tuberculosis*. *Pathog. Dis.* 76:fty039. 10.1093/femspd/fty039 29788125

[B69] Tandon H. Sharma A. Sandhya S. Srinivasan N. Singh R. (2019a). *Mycobacterium tuberculosis* Rv0366c-Rv0367c encodes a non-canonical PezAT-like toxin-antitoxin pair. *Sci. Rep.* 9:1163. 10.1038/s41598-018-37473-y 30718534 PMC6362051

[B70] Tandon H. Sharma A. Wadhwa S. Varadarajan R. Singh R. Srinivasan N. (2019b). Bioinformatic and mutational studies of related toxin-antitoxin pairs in *M. tuberculosis* predict and identify key functional residues. *J. Biol. Chem.* 294 9048–9063. 10.1074/jbc.RA118.006814 31018964 PMC6556569

[B71] Teale F. W. Weber G. (1957). Ultraviolet fluorescence of the aromatic amino acids. *Biochem. J.* 65 476–482. 10.1042/bj0650476 13412650 PMC1199900

[B72] Tiwari P. Arora G. Singh M. Kidwai S. Narayan O. P. Singh R. (2015). MazF ribonucleases promote *Mycobacterium tuberculosis* drug tolerance and virulence in guinea pigs. *Nat. Commun.* 6:6059. 10.1038/ncomms7059 25608501

[B73] Tiwari P. Gosain T. P. Singh M. Sankhe G. D. Arora G. Kidwai S. (2019). Inorganic polyphosphate accumulation suppresses the dormancy response and virulence in *Mycobacterium tuberculosis*. *J. Biol. Chem.* 294 10819–10832. 10.1074/jbc.RA119.008370 31113860 PMC6635458

[B74] Trapnell C. Williams B. A. Pertea G. Mortazavi A. Kwan G. van Baren M. J. (2010). Transcript assembly and quantification by RNA-Seq reveals unannotated transcripts and isoform switching during cell differentiation. *Nat. Biotechnol.* 28 511–515. 10.1038/nbt.1621 20436464 PMC3146043

[B75] van Kessel J. C. Marinelli L. J. Hatfull G. F. (2008). Recombineering mycobacteria and their phages. *Nat. Rev. Microbiol.* 6 851–857. 10.1038/nrmicro2014 18923412 PMC3503148

[B76] von Mering C. Huynen M. Jaeggi D. Schmidt S. Bork P. Snel B. (2003). STRING: a database of predicted functional associations between proteins. *Nucleic Acids Res.* 31 258–261. 10.1093/nar/gkg034 12519996 PMC165481

[B77] Wang X. Lord D. M. Cheng H. Y. Osbourne D. O. Hong S. H. Sanchez-Torres V. (2012). A new type V toxin-antitoxin system where mRNA for toxin GhoT is cleaved by antitoxin GhoS. *Nat. Chem. Biol.* 8 855–861. 10.1038/nchembio.1062 22941047 PMC3514572

[B78] Winther K. Tree J. J. Tollervey D. Gerdes K. (2016). VapCs of *Mycobacterium tuberculosis* cleave RNAs essential for translation. *Nucleic Acids Res.* 44 9860–9871. 10.1093/nar/gkw781 27599842 PMC5175351

[B79] Winther K. S. Brodersen D. E. Brown A. K. Gerdes K. (2013). VapC20 of *Mycobacterium tuberculosis* cleaves the sarcin-ricin loop of 23S rRNA. *Nat. Commun.* 4:2796. 10.1038/ncomms3796 24225902

[B80] Winther K. S. Gerdes K. (2011). Enteric virulence associated protein VapC inhibits translation by cleavage of initiator tRNA. *Proc. Natl. Acad. Sci. U.S.A.* 108 7403–7407. 10.1073/pnas.1019587108 21502523 PMC3088637

[B81] Zaveri A. Wang R. Botella L. Sharma R. Zhu L. Wallach J. B. (2020). Depletion of the DarG antitoxin in *Mycobacterium tuberculosis* triggers the DNA-damage response and leads to cell death. *Mol. Microbiol.* 1–12. 10.1111/mmi.14571 32634279 PMC7689832

[B82] Zhang Y. Zhang J. Hoeflich K. P. Ikura M. Qing G. Inouye M. (2003). MazF cleaves cellular mRNAs specifically at ACA to block protein synthesis in *Escherichia coli*. *Mol. Cell.* 12 913–923. 10.1016/S1097-2765(03)00402-7 14580342

[B83] Zhu L. Sharp J. D. Kobayashi H. Woychik N. A. Inouye M. (2010). Noncognate *Mycobacterium tuberculosis* toxin-antitoxins can physically and functionally interact. *J. Biol. Chem.* 285 39732–39738. 10.1074/jbc.M110.163105 20876537 PMC3000954

