# Supplementary material for: VapC21 Toxin Contributes to Drug-Tolerance and Interacts With Non-cognate VapB32 Antitoxin in Mycobacterium tuberculosis
Source: Front Microbiol. 2020 Sep 11;11:2037. doi: 10.3389/fmicb.2020.02037 (PMC7517352; doi:10.3389/fmicb.2020.02037)
Supplement: FIGURE S1 — Deletion of vapC21 does not affect growth of M. tuberculosis in different conditions. (A,B) Schematic representation for the construction of ΔvapC21 strain of M. tuberculosis Erdman. (A) The open reading frame for vapC21 was replaced with the hygromycin resistance gene in the ΔvapC21 mutant strain (MT1 and MT2), using temperature sensitive mycobacteriophages. (B) The replacement of vapC21 with the hygromycin resistance gene in the mutant strain was confirmed by Southern blot. Solid line (P) depicts the region for hybridization of probes. WT represent wild type strain of M. tuberculosis. (C) The colony morphology and biofilm formation of the wild type and ΔvapC21 mutant strain was determined as described in section “Materials and Methods.” (D–G) For stress experiments, early-log phase cultures (OD600nm ∼ 0.2) were exposed to different stress conditions such as oxidative stress (D), nitrosative stress (E), nutrient starvation (F), and 2.5 mg/ml lysozyme (G) as described in section “Materials and Methods.” The data shown in panels (D–G) are mean ± SE. obtained from triplicate samples and represents two independent experiments. [file Presentation_1.pdf]

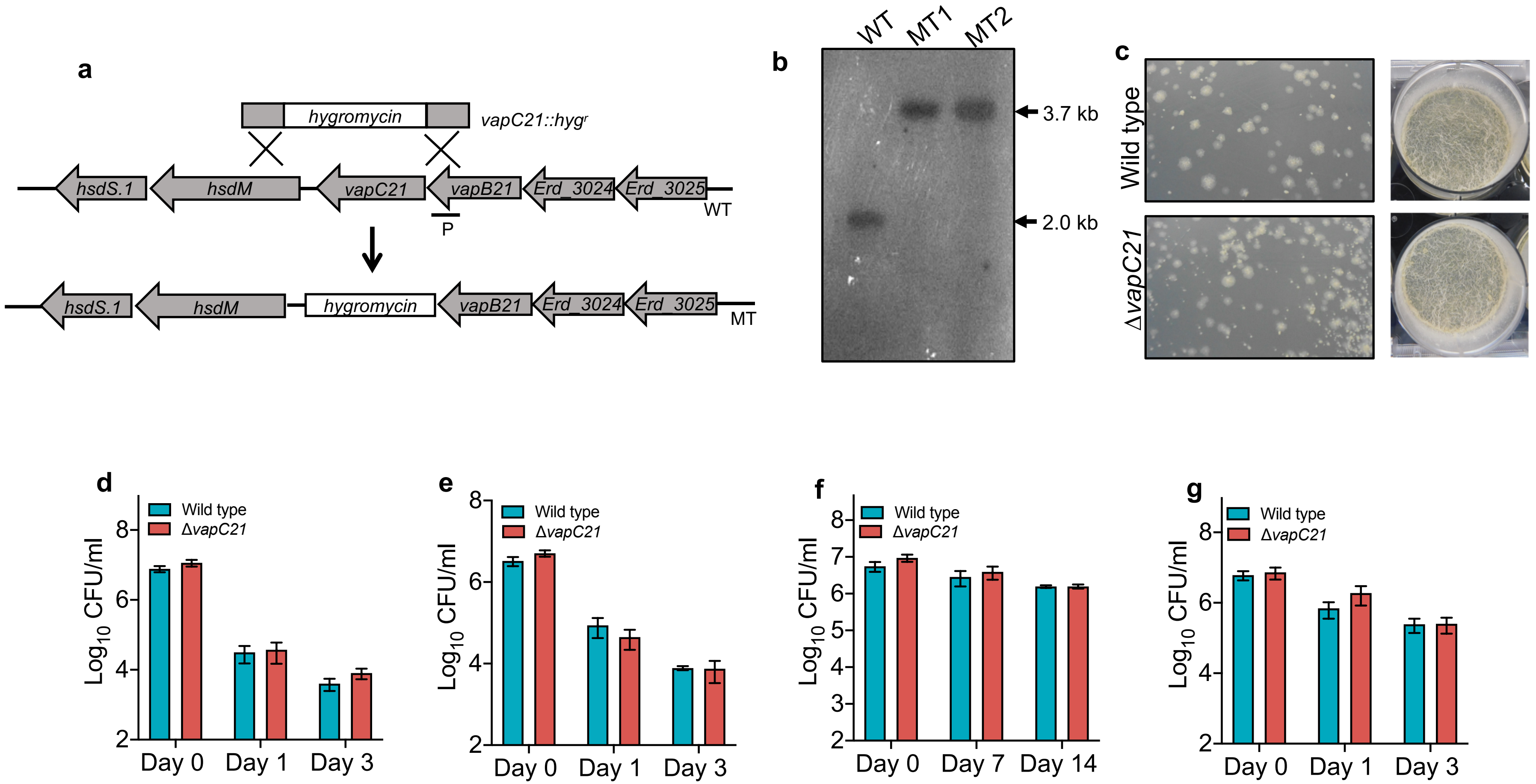

Sharma et al., Figure S1

a

	Rifampicin	Isoniazid	Levofloxacin	Ethambutol
Wild type	0.025 μM	0.78 μM	0.78 μM	3.125 μM
Δ*vapC21*	0.025 μM	0.78 μM	0.78 μM	3.125 μM

b

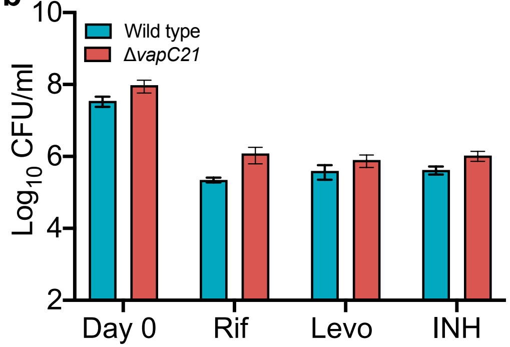

Sharma et al., Figure S2

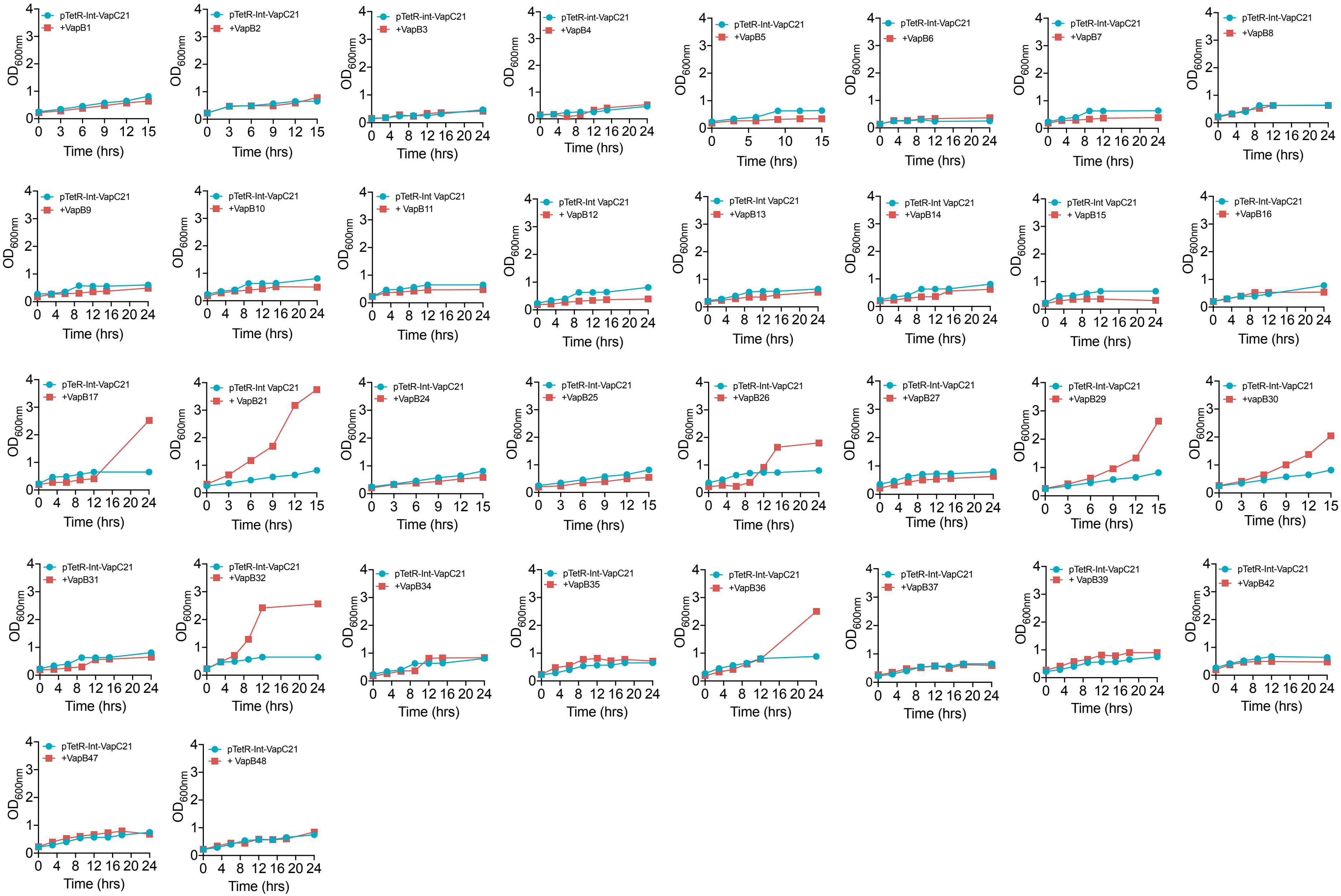

Sharma et al., Figure S3
