# Supplementary material for: VapC21 Toxin Contributes to Drug-Tolerance and Interacts With Non-cognate VapB32 Antitoxin in Mycobacterium tuberculosis
Source: Front Microbiol. 2020 Sep 11;11:2037. doi: 10.3389/fmicb.2020.02037 (PMC7517352; doi:10.3389/fmicb.2020.02037)
Supplement: TABLE S1 — List of strains and plasmids used in the present study. [file Table_1.DOCX]

| **Strains** | **Description** | **References** |
| --- | --- | --- |
| *M. tuberculosis* Erdman | Virulent strain of *Mycobacterium tuberculosis* | Kind gift from Dr. Anil K Tyagi |
| *M. Smegmatis* mc^2^ 155 | Non-pathogenic fast growing mycobacteria | Kind gift from Dr. Anil K Tyagi |
| *ΔvapC21* | Rv 2757c mutant strain of *M. tuberculosis Erdman* | This Work |
| *ΔvapC21*-CT | Rv2757c complemented strain of *M. tuberculosis Erdman* | This Work |
| *M. tuberculosis* H_37_Rv | Parental strain of *M. tuberculosis* used for overexpression RNA-seq | Kind gift from Dr. Anil K Tyagi |
| BL-21 (lambda DE3, plysE) | *E.coli* strain used for protein expression and purification | This Work |
| **Plasmids** |  |  |
| pTetR | anhydrotetracycline based mycobacterial expression | Agarwal et al., 2018 |
| pTetR-*vapC21* | pTetR harboring Rv2757c from *M. tuberculosis* | Agarwal et al., 2018 |
| pTetR-Int | anhydrotetracycline based mycobacterial expression integrative vector | Agarwal et al., 2018 |
| pTetR-Int *vapC21* | pTetR-Int harboring Rv2757c from *M. tuberculosis* | This Work |
| pLam12 | Acetamide inducible episomal mycobacterial expression vector | Van Kessel et al., 2007 |
| pLam12 *vapB1* | pLam12 harbouring Rv0064a from *M. tuberculosis* | This Work |
| pLAm12 *vapB2* | pLam12 harbouring Rv0300c from *M. tuberculosis* | This Work |
| pLam12 *vapB3* | pLam12 harbouring Rv0550c from *M. tuberculosis* | This Work |
| pLam12 *vapB4* | pLam12 harbouring Rv0596c from *M*. *tuberculosis*. | This Work |
| pLam12 *vapB5* | pLam12 harbouring Rv0626 from *M. tuberculosis* | This Work |
| pLam 12 *vapB6* | pLam12 harbouring Rv0657c from *M. tuberculosis* | This Work |
| pLam12 *vapB7* | pLam12 harbouring Rv0662c from *M. tuberculosis* | This Work |
| pLam12 *vapB8* | pLam12 harbouring Rv0664 from *M. tuberculosis* | This Work |
| pLam12 *vapB9* | pLam12 harbouring Rv0959A from *M. tuberculosis* | This Work |
| pLam12 *vapB10* | pLam12 harbouring Rv1398c from *M. tuberculosis* | This Work |
| pLam12 *vapB11* | pLam12 harbouring Rv1560 from *M. tuberculosis* | This Work |
| pLam12 *vapB12* | pLam12 harbouring Rv1721c from *M. tuberculosis* | This Work |
| pLam12 *vapB13* | pLam12 harbouring Rv1839c from *M. tuberculosis* | This Work |
| pLam12 *vapB14* | pLam12 harbouring Rv1952 from *M. tuberculosis* | This Work |
| pLam12 *vapB15* | pLam12 harbouring Rv2009 from *M. tuberculosis* | This Work |
| pLam12 *vapB16* | pLam12 harbouring Rv2231B from *M. tuberculosis* | This Work |
| pLam12 *vapB17* | pLam12 harbouring Rv2526 from *M. tuberculosis* | This Work |
| pLam12 *vapB21* | pLam12 harbouring Rv2758c from *M. tuberculosis* | This Work |
| pLam12 *vapB24* | pLam12 harbouring Rv0239 from *M. tuberculosis* | This Work |
| pLam12 *vapB25* | pLam12 harbouring Rv0277A from *M. tuberculosis* | This Work |
| pLam12 *vapB26* | pLam12 harbouring Rv0581 from *M. tuberculosis* | This Work |
| pLam12 *vapB27* | pLam12 harbouring Rv0599c from *M. tuberculosis* | This Work |
| pLam12 *vapB29* | pLam12 harbouring Rv0616A from *M. tuberculosis* | This Work |
| pLam12 *vapB30* | pLam12 harbouring Rv0623 from *M. tuberculosis* | This Work |
| pLam12 *vapB31* | pLam12 harbouring Rv0748 from *M. tuberculosis* | This Work |
| pLam12 *vapB32* | pLam12 harbouring Rv1113 from *M. tuberculosis* | This Work |
| pLam12 *vapB34* | pLam12 harbouring Rv1740 from *M. tuberculosis* | This Work |
| pLam12 *vapB35* | pLam12 harbouring Rv1962a from *M. tuberculosis* | This Work |
| pLam12 *vapB36* | pLam12 harbouring Rv1982a from *M. tuberculosis* | This Work |
| pLam12 *vapB37* | pLam12 harbouring Rv2104c from *M. tuberculosis* | This Work |
| pLam12 *vapB39* | pLam12 harbouring Rv2530A from *M. tuberculosis* | This Work |
| pLAm12 *vapB42* | pLam12 harbouring Rv2760c from *M. tuberculosis* | This Work |
| pLam12 *vapB47* | pLam12 harbouring Rv3407 from *M. tuberculosis* | This Work |
| pLam12 *vapB48* | pLAm12 harbouring Rv3697A from *M. tuberculosis* | This Work |
| pETDuet-1 *vapBC21* | pETDuet-1 harbouring Rv2757c - Rv2758c (VapBC21) complex from  *M. tuberculosis* | This Work |
| pET15b *vapC21* | pET15b harbouring Rv2757c from  *M. tuberculosis* | This Work |
| pET15b *vapB3* | pET15b harbouring Rv0550c from  *M. tuberculosis* | This Work |
| pET15b *vapB4* | pET15b harbouring Rv0596c from  *M. tuberculosis* | This Work |
| pET15b *vapB21* | pET15b harbouring Rv2758c from  *M. tuberculosis* | This Work |
| pET15b *vapB26* | pET15b harbouring Rv0581 from  *M. tuberculosis* | This Work |
| pET15b *vapB32* | pET15b harbouring Rv1113 from  *M. tuberculosis* | This Work |

**Table S1:** List of strains and plasmids used in the present study.
